# Subnational variation for care at birth in Tanzania: is this explained by place, people, money or drugs?

**DOI:** 10.1186/s12889-016-3404-3

**Published:** 2016-09-12

**Authors:** Corinne E. Armstrong, Melisa Martínez-Álvarez, Neha S. Singh, Theopista John, Hoviyeh Afnan-Holmes, Chris Grundy, Corrine W. Ruktanochai, Josephine Borghi, Moke Magoma, Georgina Msemo, Zoe Matthews, Gemini Mtei, Joy E. Lawn

**Affiliations:** 1 Centre for Maternal, Adolescent, Reproductive and Child Health, London School of Hygiene & Tropical Medicine, London, WC1E 7HT UK; 2 Evidence for Action, Dar es Salaam, Tanzania; 3 World Health Organization, 1 Luthuli Street, PO Box 9292, Dar es Salaam, Tanzania; 4 Independent consultant, London, UK; 5 Department of Infectious Disease Epidemiology, London School of Hygiene & Tropical Medicine, London, WC1E 7HT UK; 6 Department of Geography & Environment, University of Southampton, Highfield, Southampton, SO17 1BJ UK; 7 Ministry of Health and Social Welfare, Dar es Salaam, Tanzania; 8 Division of Social Statistics and Demography & Centre for Global Health, Population, Poverty and Policy, Faculty of Social and Human Sciences, University of Southampton, Highfield, Southampton, UK; 9 Ifakara Health Institute, Dar es Salaam, Tanzania

**Keywords:** Health systems, Health financing, Health workforce, Childbirth, Quality of care, Newborn health, Maternal health, Tanzania

## Abstract

**Background:**

Tanzania achieved the Millennium Development Goal for child survival, yet made insufficient progress for maternal and neonatal survival and stillbirths, due to low coverage and quality of services for care at birth, with rural women left behind. Our study aimed to evaluate Tanzania’s subnational (regional-level) variations for rural care at birth outcomes, i.e., rural women giving birth in a facility and by Caesarean section (C-section), and associations with health systems inputs (financing, health workforce, facilities, and commodities), outputs (readiness and quality of care) and context (education and GDP).

**Methods:**

We undertook correlation analyses of subnational-level associations between health system inputs, outputs, context, and rural care at birth outcomes; and constructed implementation readiness barometers using benchmarks for each health system input indicator. We used geographical information system (GIS) mapping to visualise subnational variations in care at birth for rural women, with a focus on service availability and readiness, and collected qualitative data to investigate financial flows from national to council level to understand variation in financing inputs.

**Results:**

We found wide subnational variation for rural care at birth outcomes, health systems inputs, and contextual indicators. There was a positive association between rural women giving birth in a facility and by C-section; maternal education; workforce and facility density; and quality of care. There was a negative association between these outcomes and proportion of all births to rural women, total fertility rate, and availability of essential commodities at facilities. Per capita recurrent expenditure was positively associated with facility births (correlation coefficient = 0.43; *p* = 0.05) but not with C-section. Qualitative results showed that the health financing system is complex and insufficient for providing care at birth services. Bottlenecks for care at birth included low density of health workers, poor availability of essential commodities, and low health financing in Lake and Western Zones.

**Conclusions:**

No region meets the benchmarks for the four health systems building blocks including health finance, health workforce, health facilities, and commodities. Strategies for addressing health system inequities, including overall increases in health expenditure, are needed in rural populations and areas of highest unmet need for family planning to improve coverage of care at birth for rural women in Tanzania.

**Electronic supplementary material:**

The online version of this article (doi:10.1186/s12889-016-3404-3) contains supplementary material, which is available to authorised users.

## Background

As the Millennium Development Goals (MDGs) era transitions to the Sustainable Development Goals (SDGs) era, a major unfinished task relates to the annual number of nearly six million maternal and neonatal deaths and stillbirths globally [1–5], the majority of these occurring around the time of birth [6]. Despite achieving MDG 4 for child survival, Tanzania has made insufficient progress for maternal and newborn survival and stillbirths prevention [7]. Tanzania ranks in the top ten countries globally in 2015 for the number of stillbirths (47,060) [8] and newborn deaths (39,000) [1], in addition to 7900 maternal deaths annually [2].

Countdown to 2015 (Countdown) is a multi-partner initiative that tracks progress in reproductive, maternal, newborn and child health (RMNCH) for the 75 highest burden countries [9]. Countdown’s Tanzania case study results showed that coverage gaps and wide inequities persist in family planning and care at birth, especially for rural women, with quality of care highlighted as a major challenge [7]. Use of modern contraceptive methods is low in the Western and Lake Zones (both 15 %) with high unmet need for family planning (26 and 33 % respectively) [7]. In 2006–2010, 84 % of urban births occurred in a health facility and 10 % by Caesarean section (C-section), compared with only 44 % of rural births in a facility and 3 % by C-section [7].

About half of Tanzania’s mothers give birth in a health facility, with only marginal increases over the last 25 years [7], and despite 96 % receiving antenatal care (ANC) at least once [10], few return for the recommended four ANC visits [11]. Care at birth service provision in Tanzania is operationalised through a tiered structure where basic services are available at dispensaries, while health centres and hospitals provide services for births including emergency obstetric care by referral.

Tanzania has developed comprehensive national strategies [12] for maternal and newborn health (MNH) that directly address the need to improve coverage and equity of care at birth services, and a nascent mechanism to measure subnational progress in its 25 regions operating as administrative subdivisions (Additional file 1), with an RMNCH scorecard [13]. However, further in-depth analyses of subnational data are needed to target resources effectively [14]. Most analyses of health system readiness to date in Tanzania and other lower- and middle-income countries focus on national level data [7, 15], have a small subnational geographical focus [16, 17], or do not relate essential inputs (e.g., financial, infrastructural and human resources) to disparities in outputs and outcomes within the health system [18–21].

**Fig. 1:**
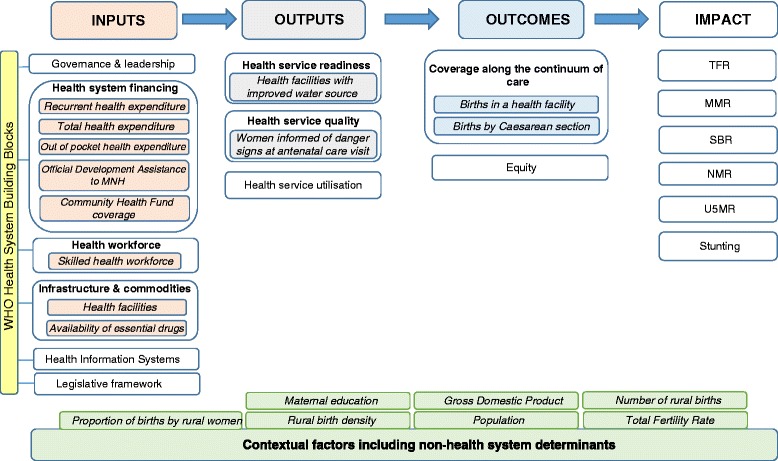
Health systems evaluation framework for Countdown to 2015 case studies

To close the inequity gap for rural women in Tanzania, and to reduce disparities between regions, subnational analyses are required for financial and human resource distribution, commodities, infrastructure, and utilisation to inform accelerated strategies [14, 22]. Our evaluation framework (Fig. 1) provides an overview of the inputs to a health system to provide quality services for care at birth. We adapted this framework to focus on MNH, based on existing literature on implementation strength and resource allocation [16]. We investigate why rural women in Tanzania are left behind [7]; specifically, to assess what we know about gaps in facilities, financing, health workforce, commodities and demand for services, and what the remaining challenges are.

## Aims and objectives

This study used mixed methods to evaluate Tanzania’s subnational (at the ‘mkoa’, or regional level) variations in care at birth outcomes (i.e., rural women giving birth in a facility and by C-section) and associations with inputs according to the health systems building blocks [23]. Specific objectives were as follows:

1. ***To describe the situation in 21 regions*** of mainland Tanzania by using nine tracer indicators related to care at birth for each of the health system building blocks - financial and human resource distribution, commodities and infrastructure, health service quality and utilisation - as a measure of health system readiness.
2. ***To undertake a correlation analysis to examine subnational level associations*** between these input health systems tracer indicators with outputs (quality of care) and outcomes, measured as births in a facility and by C-section.
3. ***To use geographical information system (GIS) mapping techniques to visualise subnational variation for care at birth outcomes*** with barometers of health system readiness.
4. ***To explore qualitatively the governance and management of health financial flows*** from national to council level to better understand subnational variation in financial allocation.

## Methods

### Overview

Our analysis used a health systems evaluation framework [22] modified to reflect care at birth services (Fig. 1), which outlines the essential components within each building block of the health system (inputs, outputs, outcomes and impact) tailored to care at birth. We adapted this health systems framework within the context of the national level Tanzania Countdown case study [7], incorporating the same outcome measures for this second analysis, and utilising best available data for the health systems building blocks.

### Objective 1. Describe the situation in 21 regions of mainland Tanzania by using tracer indicators related to care at birth for each of the health system building blocks

We describe contextual data for each region in Tanzania according to a priori known associations with care at birth [24, 25] We extracted 2012 census population data [26]. Subnational gross domestic product (GDP) was abstracted from the National Accounts 2000–2010 [27] and estimated per capita with census data [26]. The proportion of women with complete primary education or higher was abstracted from 2010 DHS data [10]. United Nations adjusted births and pregnancies data (2010) were obtained from a previous analysis [28] (Additional file 1) - we extracted total fertility rate (TFR), number of live births, number of rural births, proportion of all births by rural women, birth density (mean births per square kilometre) and rural birth density.

We collected the best available data at subnational level in mainland Tanzania corresponding with each health system building block in the evaluation framework (Fig. 1), selecting proxy indicators according to data availability and reliability (Table 1).

**Table 1:** Health system tracer indicators and data sources related to coverage of care at birth for rural women in Tanzania

Contextual factors		Inputs			Outputs			Outcomes		
Tracer indicator	Source	Building block	Tracer Indicator	Source	Building block	Tracer Indicator	Source	Building block	Tracer Indicator	Source
Total Population (2012)	2012 census [26]	Governance and leadership	Qualitative analysis - no standardised quantitative data for assessment		Health Service Quality	% women informed of danger signs at ANC	2010 DHS Report [10]	Coverage Along the Continuum of Care	Proportion of births by rural women by Caesarean section	Original analysis using 2010 DHS [10]
GDP per capita	Original analysis using National Accounts 2000-2010 [27]	Health System Financing	Per capita recurrent health expenditure	PMO-RALG Local Government Financial Report (FY 2012/13) [29]	Health Service Utilisation	No data available at regional level			Proportion of births by rural women in a health facility	Original analysis using 2010 DHS [10]
Maternal education	Original analysis using 2010 DHS [10]		Per capital total health expenditure	PMO-RALG Local Government Financial Report (FY 2012/13) [29]	Health Service Readiness	% facilities with improved water source	Tanzania Service Provision Assessment Survey 2014–15 [38]	Equity	Cross-cutting thematic analysis - regional equity	
Proportion of births by rural women	Original analysis using worldpop data [28]		Out of Pocket health expenditure	Household Budget Survey 2007 [30]						
Number of births	Original analysis using worldpop data [28]		Community Health Fund coverage	Fact Sheet Inside NHIF 2001/02 to 30th June 2013 NHIF [33]						
Rural Birth Density	Original analysis using worldpop data [28]		Official Development Assistance to MNH	Partners Mapping and Resource Tracking 2013/14 with personal communication [31]						
Total Fertility Rate	Original analysis using worldpop data [28]	Health Workforce	Skilled health workforce density	Original analysis using 2012/13 HRH Country Profile [34] and Census [26]						
		Infrastructure and Commodities	Health facility density - health centres & hospitals	Service Provision Assessment Survey 2014 data provided by personal communication [36]						
			Availability of essential drugs	National RMNCH scorecard from HMIS data (quarter 4 of 2014)						
		Health Information Systems	No data accessible							
		Legislative framework	Not applicable at regional level							

#### Health financing input

Financial input indicators of recurrent (government) health expenditure from 2012/13 [29], 2007 average annual household Out of Pocket health expenditure (OOP) [30], and 2013/14 Official Development Assistance for MNH (ODA) [31] (Table 1) were obtained (Additional file 1). We calculated per capita expenditures [26], converting to 2013 USD using Bank of Tanzania conversion rates and World Bank deflators [32]. Community Health Fund (CHF) – a community based health insurance scheme - 2013 coverage data were obtained from National Health Insurance Fund (NHIF) reports [33] (Table 1).

#### Health workforce input

Health workforce density data for those cadres involved in MNH service provision were derived from the human resources data in the health country profile [34]. We assumed that Assistant Medical Officer, Assistant Nursing Officer, Medical Consultant, Medical Doctor, Medical Specialist, Nurse, Nurse Midwives, and Nursing Officer are capable of providing skilled birth care [35]. Health workforce densities were reported as a ratio per 10,000 capita and per 10,000 births.

#### Health facilities input

We estimated the total number of facilities providing basic and comprehensive emergency care at birth (health centres and hospitals) in each subnational region from data provided by Ministry of Health and Social Welfare (MoHSW) – now the Ministry of Health, Community Development, Gender, Elderly and Children [36]. Health facility densities were reported as a ratio per 10,000 capita and per 10,000 births.

#### Commodities input

The proportion of all facilities with no stockouts of essential commodities (Additional file 1) were extracted from the 2014 quarter four national RMNCH scorecard using Health Management Information System (HMIS) data [37] as a measure of commodities supply.

#### Quality of care and health service readiness output

We used the proportion of women who attended ANC and subsequently recalled being informed of signs of pregnancy complications, as a proxy for quality of care [10]. For health service readiness we used percentage of all health facilities with improved water source (Additional file 1) from the Tanzania Service Provision Assessment Survey 2014-15 [38], in accordance with recent evidence associating water and sanitation with maternal mortality [39, 40]. Reliable health service utilisation data were not available at subnational level.

#### Coverage of care at birth outcome

Building on the national Countdown case study [7], we used 2010 DHS data [10] to calculate the proportion of all births (inclusive of C-section) occurring in a health facility (hospital, health centre or dispensary) – a proxy for our outcome of skilled birth attendance [41] - and the percentage of births by C-section – a proxy for our outcome of emergency obstetric care [42] (Additional file 1). Both outcomes are self-reported by women.

We restricted analyses of outcome indicators to births by rural women based upon findings of the Countdown country case study: rural/urban disparity is the strongest inequity [7]. Additionally, 70 % of Tanzania’s population is rural [10], and the literature illustrates that urban women generally access facilities for births (82 %) [7, 10].

In 2012, four new subnational regions were demarcated (Additional file 1); thus we recalculated district-level data for several indicators (total population, total births, recurrent expenditure, total expenditure, CHF, ODA, health workforce, health facilities, and commodities supply) to ensure consistency with the subnational boundaries in place at the time of the 2010 DHS (Additional file 1) [10].

### Objective 2. Undertake a correlation analysis to examine subnational level associations between health systems inputs, outputs and outcomes

Bivariate correlation analyses were performed across all levels of the evaluation framework, using Stata 13.1. Less than 5 % chance was considered statistically significant. A correlation coefficient (CC) of greater than 0.80 was considered a very strong association, 0.60–0.79 a strong association, 0.40–0.59 a moderate association, and <0.40 a weak association adopted from recent literature [43] and considered within the context of this analysis.

### Objective 3. Use GIS mapping techniques to visualise subnational variation for care at birth outcomes with barometers of health system readiness

Choropleth and proportional maps were generated using Arc GIS 10.3 software [44] to illustrate subnational variations in: (i) rural birth density; (ii) births by rural women in a health facility; (iii) births by rural women by C-section; (iv) per capita recurrent expenditure; (v) health workforce density; (vi) health facility density; and (vii) health facilities availability of tracer drugs. Health facility and health workforce data were mapped using both population and births as denominators, taking into account recent recommendations from Gabrysch et al. [45].

Implementation readiness barometers developed by the Countdown Health Systems and Policies Technical Working Group [46] were drafted for each Tanzanian region based upon the WHO health system building blocks, to be overlaid with choropleth maps showing variation in proportion of births by rural women a) in a health facility and, b) by C-section. This approach was applied to identify “good” and “poor” performing regions and to assess subnational variation in care at birth, with a focus on service availability and readiness.

Implementation readiness barometers were constructed using data from HMIS [37], Human Resources for Health Country Profile (2012/13) [34], 2012 Census [26], the Prime Minister’s Office for Regional Administration and Local Government (PMO-RALG, now the President’s Office for Regional Administration and Local Government) Financial Reports database [29], DHS 2010 [10], and facility data provided by MoHSW [36] for the following interlinked indicators based on four WHO health system building blocks (health financing, workforce, commodities, and facilities): (i) per capita recurrent expenditure [29]; (ii) skilled health workforce density per 10,000 population [26, 34]; (iii) availability of tracer drugs at health facilities [37]; and (iv) health facilities per 10,000 population [26, 36].

**Fig. 2:**
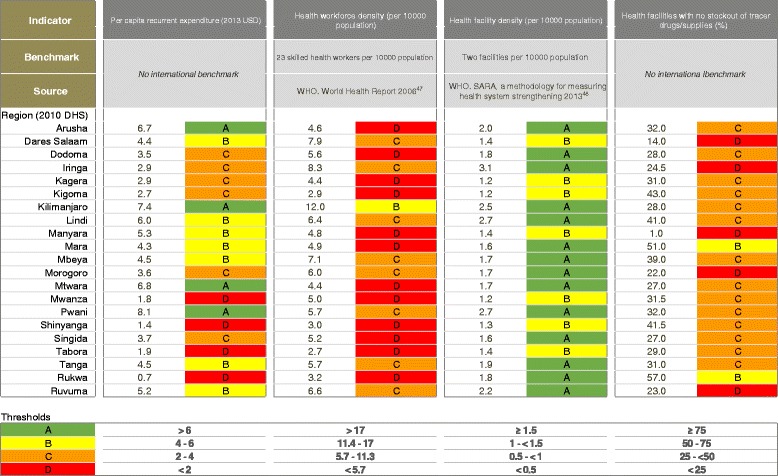
Benchmarks categorising health systems data to construct implementation readiness scores for regions in Tanzania

Applying methodology developed by Countdown [46], data for each health systems indicator were categorised according to proportional achievement of a benchmark, as follows: (i) green: ≥ 75 %; (ii) yellow: 50– <75 %; (iii) orange: 25– <50 %; (iv) red: <25 % (Fig. 2). International benchmarks were used for categorising health workforce and health facilities data [47, 48] (Fig. 2)*.* No international benchmarks exist for per capita recurrent expenditure or commodities availability. Thus, we allocated four groups representing the diversity in funding levels and categorised as green the subnational regions with the highest expenditure levels. We used ≥75 % as a benchmark for available tracer drugs.

### Objective 4. Explore qualitatively the governance and management of health financial flows

The aim of the interviews was to explore the budget and decision-making process to understand resource flows and identify potential bottlenecks at different levels of the health financing system, and across different types of health expenditure. Twenty-two purposively sampled semi-structured interviews were undertaken with stakeholders from MoHSW, PMO-RALG, representatives of development partners and regional and council health management teams. A semi-structured interview guide was developed during a pre-fieldwork site visit. Interviews were conducted in one region and two districts between April and July 2012, in English, face-to-face and each lasted approximately one hour. All the interviews were conducted and analysed by one author (MMA). Where respondents agreed, interviews were recorded and transcribed; otherwise, notes were taken during interviews and immediately typed up. Data were analysed using thematic analysis [49], involving several stages: data familiarisation, code generation, search and review themes and defining themes.

## Results

### Objective 1. Describe the situation in 21 regions of mainland Tanzania by using tracer indicators related to care at birth for each of the health system building blocks

**Fig. 3:**
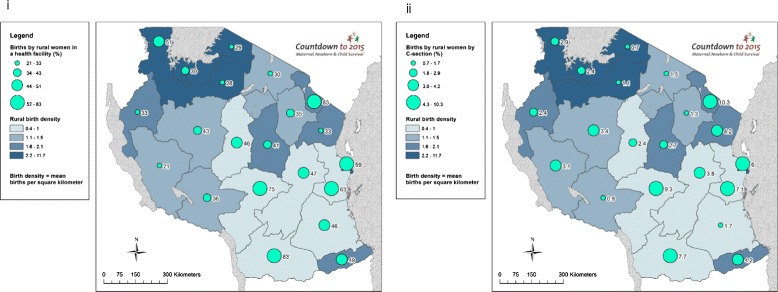
Tanzania subnational maps of coverage of care at birth for rural women compared to rural birth density: (i) Births by rural women in a health facility (%); and (ii) Births by rural women by Caesarean section (%)

Large regional level variations are seen across the contextual data. Population ranges from under one million in Lindi region to over four million in Dar es Salaam, and GDP per capita ranges from TZS 425,786 in Kigoma region to TZS 1,243,774 in Dar es Salaam (Additional file 1). Large inequities exist in maternal education; only 47 % (95 % CI: 35–59 %) of women in Tabora had completed primary school education or higher compared to 91 % (95 % CI: 87–94 %) in Kilimanjaro (Additional file 1). Rural birth density varied widely; from 0.42 births per km^2^ in Lindi to 11.72 births per km^2^ in the mainly urban Dar es Salaam, and high rural birth densities are seen in rural remote regions of Mara (3.51 births per km^2^) and Shinyanga (3.44 births per km^2^) (Additional file 1). TFR ranges from three births per woman in Dar es Salaam to eight in Rukwa (Additional file 1). Regions with highest rural birth density are in general not matched with greater births by rural women in a health facility (Fig. 3)]. The Lake Zone regions have highest rural birth densities and among the lowest proportion of facility births (Mara = 29 %; Mwanza = 39 %; Shinyanga = 28 %) and C-section (Kagera = 2.9 %; Mara = 0.7 %; Mwanza = 2.4 %; Shinyanga = 1.1 %).

#### Health financing input

**Fig. 4:**
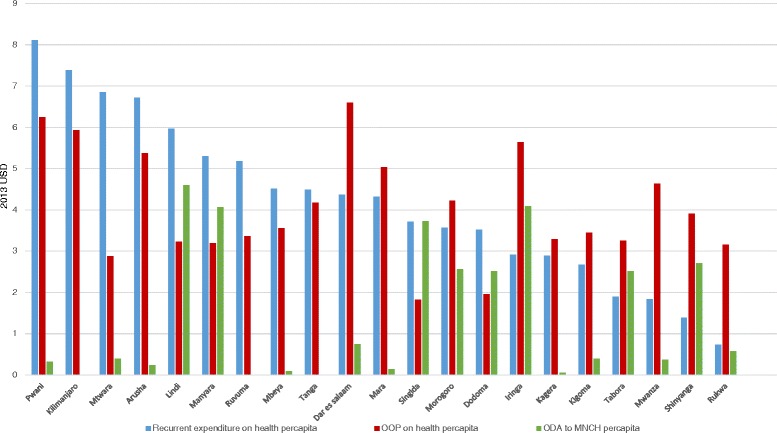
Sources of financial inputs for health per region in Tanzania (USD)

Recurrent per capita expenditure was unequally distributed between regions; Pwani received from government USD 8.1 per capita, while Rukwa only received USD 0.7 per capita (Additional file 1). Additionally, ODA per capita favoured the regions of Lindi (USD 4.6), Manyara (USD 4.1), Iringa (USD 4.1) and Singida (USD 3.7) (Fig. 4). Per capita OOP also varied; between USD 1.8 in Singida and USD 6.6 in Dar es Salaam region.

#### Health workforce, facilities, and commodities inputs

Despite large subnational discrepancies in the MNH health workforce, health workforce density in all regions is far below the WHO minimum density threshold of 23 skilled health workers per 10,000 (Additional file 1) - although the WHO threshold refers to all skilled health workers. Tabora had just under three health workers involved in MNH service provision per 10,000 population compared to Kilimanjaro with 12 – possibly a result of several factors including concentration around the national teaching hospital, more educated population, and higher standards of living (Additional file 1). Facility density per 10,000 births (health centres and hospitals) varies from approximately 17 in Kilimanjaro to just over two in Shinyanga (Additional file 1). The percentage of health facilities with available tracer drugs (Additional file 1) ranges from 1 % in Manyara to 57 % in Rukwa (Additional file 1).

#### Quality of care and health service readiness output

Quality of care varies dramatically across Tanzania. The proportion of women informed of signs of pregnancy complications at ANC ranges from 27 % in Mwanza to 80 % in Dar es Salaam (Additional file 1). Health service readiness ranged from 43 % of health facilities in Lindi with an improved water source, to 87 % in Kagera (Additional file 1).

#### Coverage of care at birth outcome

Large subnational inequities exist for both non-emergency and emergency obstetric care in Tanzania. Just 21 % (95 % CI: 12–33 %) of rural births in Rukwa take place in a health facility compared to 83 % in both Ruvuma (95 % CI: 71–91 %) and Kilimanjaro (95 % CI: 75–89 %). Less than 1 % of rural births are by C-section in Mara (0.7 %; 95 % CI: 0.2–2.6 %) and Mbeya (0.9 %; 95 % CI: 0.2–3.9 %) compared to 10 % in Kilimanjaro (10 %; 95 % CI: 6.0–17.4 %) (Additional file 1).

### Objective 2. Undertake a correlation analysis to examine subnational level associations between health systems inputs, outputs and outcomes

**Fig. 5:**
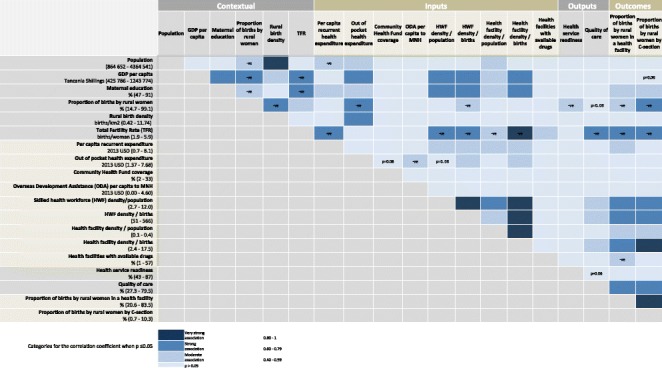
Tanzanian subnational associations between coverage of care at birth with health systems inputs, outputs and contextual indicators

Correlation analyses revealed that maternal education is moderately associated with both care at birth outcomes (CC for births in a facility = 0.58 and for births by C-section = 0.57; p-value for both outcomes = 0.01) (Fig. 5). There is a strong negative association between TFR and both care at birth outcomes (births in a facility CC = −0.70; by C-section CC = −0.68; and both *p*-values <0.01), as well as quality of care (CC = −0.66, *p* < 0.01) (Fig. 5). In addition, TFR has a strong negative association with health workforce (CC = −0.79, *p* < 0.01), maternal education (CC = −0.67, *p* < 0.01), and GDP per capita (CC = −0.64, *p* < 0.01) and a very strong negative association with facility density (C = −0.81, *p* < 0.01). A strong negative association is seen between the proportion of births to rural women and GDP per capita (CC = −0.64, *p* < 0.01), OOP (CC = −0.63, *p* < 0.01) and births by C-section (CC = −0.63, *p* < 0.01), while a moderate negative association exists between proportion of births to rural women and health service readiness (CC = −0.44, *p* = 0.04).

#### Health financing input

Per capita OOP expenditure has a negative moderate association with ODA (CC = −0.43; p-value = 0.05) but unexpectedly showed no correlation with government expenditure (Figs. 4 and 5). Importantly, we found no significant correlation between both outcomes and any of the financing indicators, with the exception of a moderate association between per capita recurrent government expenditure and percentage of births in a facility (CC = 0.43; *p* = 0.05) (Fig. 5). Interestingly, a similar association was not seen for births by C-section (CC = 0.2; *p* = 0.40) (Fig. 5).

#### Health workforce input

We found a strong association between health workforce density (by both births and population denominators) with both outcomes (CC for these analyses are between 0.69 and 0.75, *p*-values <0.01) (Fig. 5).

#### Health facilities input

Health facility density by population is moderately associated with both outcomes for care at birth. The strength of evidence and associations increased when assessing facility density by births – births in a health facility are strongly associated (CC = 0.73; *p*-value < 0.01) and births by C-section are very strongly associated (CC = 0.81; *p*-value <0.01) with facility density by births (Fig. 5).

#### Commodities input

Availability of tracer drugs has an inverse moderate association with births in a health facility (CC = −0.45; p-value = 0.04) and no association with C-section (Fig. 5).

#### Quality of care and health service readiness output

The proxy quality of care indicator is strongly associated with both outcomes measures (births in a facility: CC = 0.64; births by C-section: CC = 0.69; p-values for both outcomes < 0.01; (Fig. 5), while the health service readiness proxy showed no associations with care at birth indicators.

#### Coverage of care at birth outcome

As expected, there is evidence of a strong association between the two outcome variables (CC = 0.89; *p*-value < 0.01) (Fig. 5).

### Objective 3. Use GIS mapping techniques to visualise subnational variation for care at birth outcomes with barometers of health system readiness

#### Health financing input

**Fig. 6:**
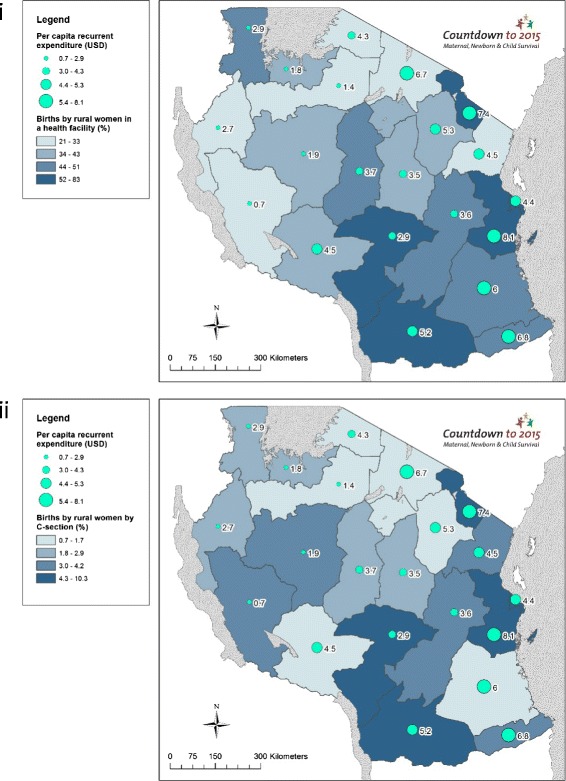
Tanzania subnational maps of coverage of care at birth for rural women compared to financial inputs: (i) Births by rural women in a health facility (%); and (ii) Births by rural women by Caesarean section (%)

We found that regions with higher levels of per capita recurrent expenditure do not necessarily have higher proportions of births in health facilities or by C-section (Fig. 6). For example, Arusha’s per capita recurrent expenditure is 6.7 USD, though only 30.5 % of its rural births take place in health facilities, and 1.5 % are by C-section. Figure 6 shows variation in spending, e.g., Southern regions have higher per capita recurrent expenditure compared to regions in Northern, Central and Western Tanzania.

#### Health workforce input

**Fig. 7:**
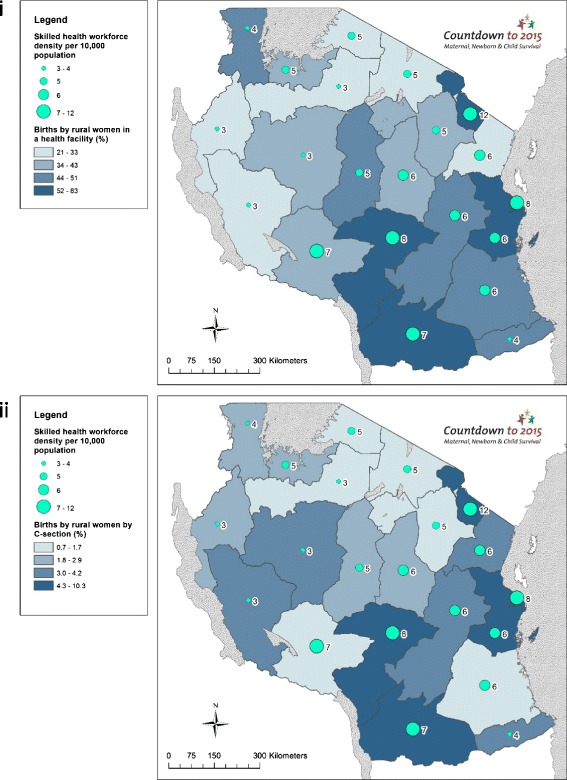
Tanzania subnational maps of coverage of care at birth for rural women compared to health workforce inputs: (i) Births by rural women in a health facility (%); and (ii) Births by rural women by Caesarean section (%)

Mapping health workforce density illustrates the higher density of skilled health workers in Southern regions compared to Northern, Western and Central Tanzania (Fig. 7). Additionally, these data highlight that with the exception of Kagera and Mbeya, there appears to be an association between health workforce density and care at birth, corroborated by our correlation analyses (Fig. 5). Kagera achieves over half of rural births taking place in facilities (51.3 %) and 2.9 % by C-section with only 4 skilled health workers per 10,000 population, while Mbeya achieves only 36 % of births by rural women in a health facility and 1.3 % by C-section with 7.1 skilled health workers per 10,000 population. Maps presenting the outcome indicators of births by rural women using the denominators recommended in Gabrysch et al. [45], i.e., density of doctors and midwives per 3600 births, are presented in the Additional file 1.

#### Health facilities input

**Fig. 8:**
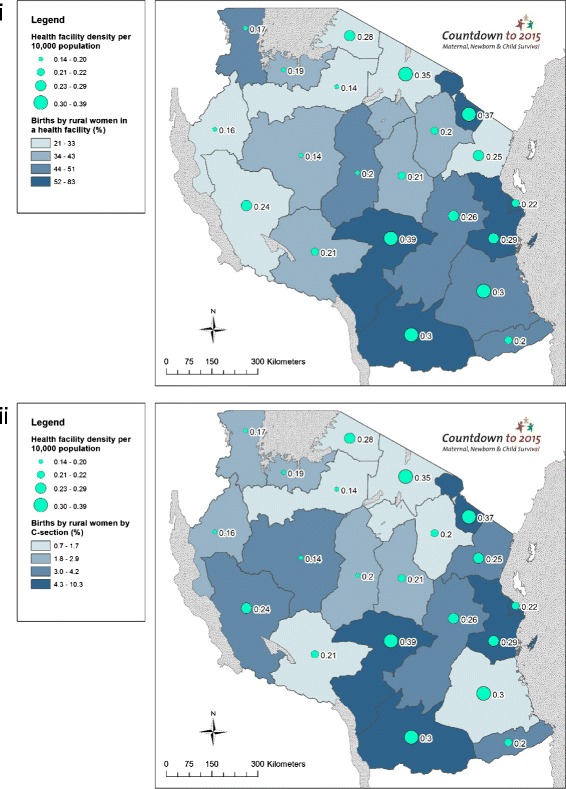
Tanzania subnational maps of coverage of care at birth for rural women compared to health facility inputs: (i) Births by rural women in a health facility (%); and (ii) Births by rural women by Caesarean section (%)

Regions with fewer births by rural women in facilities and by C-section show a lower facility density (Fig. 8). Again, Kagera stands out with a density of just 0.17 health facilities per 10,000 population, with 51 % facility births, although C-section rate is low at 2.9 %. While Arusha has a relatively high facility density (0.35 per 10,000 population) only 30.2 % of rural births take place in a facility and 1.5 % by C-section. Maps presenting the outcome indicators of births by rural women using denominator recommended in Gabrysch et al. [45], i.e., density of health facilities per 20,000 births, are presented in the Additional file 1.

#### Commodities input

**Fig. 9:**
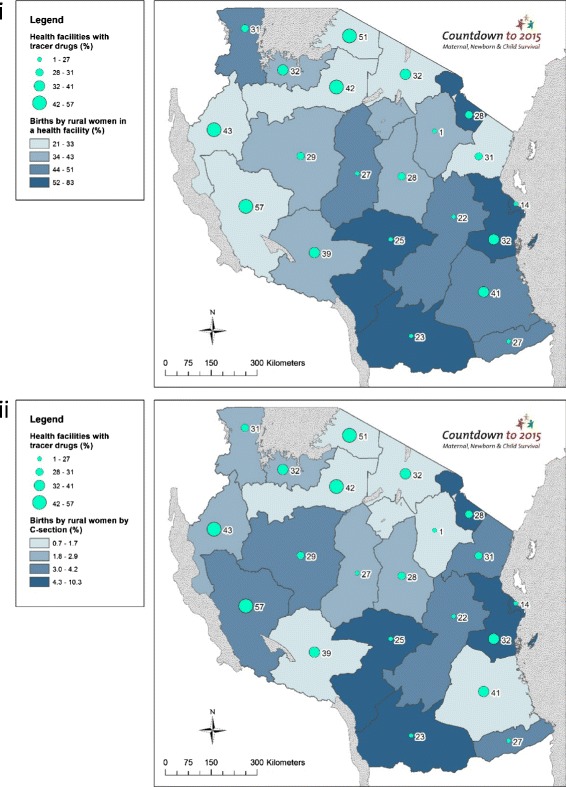
Tanzania subnational maps of coverage of care at birth for rural women compared to drugs inputs: (i) Births by rural women in a health facility (%); and (ii) Births by rural women by Caesarean section (%)

Our mapping analysis paints a mixed picture for tracer drugs availability. Rukwa - the best performing region with relatively lower proportions of facility births and C-sections - reports only 57 % availability of required tracer drugs (Fig. 9). Overall, regions with the highest proportions of rural facility births and rural birth by C-section have higher stock outs of tracer drugs, which may suggest that drugs are more diminished where women use health services. For example, 83.5 % of rural births take place in facilities in Ruvuma and 7.7 % of rural births are delivered by C-section; however, its facilities report having less than a quarter (23 %) of the required tracer drugs at its facilities.

#### Health system implementation readiness

**Fig. 10:**
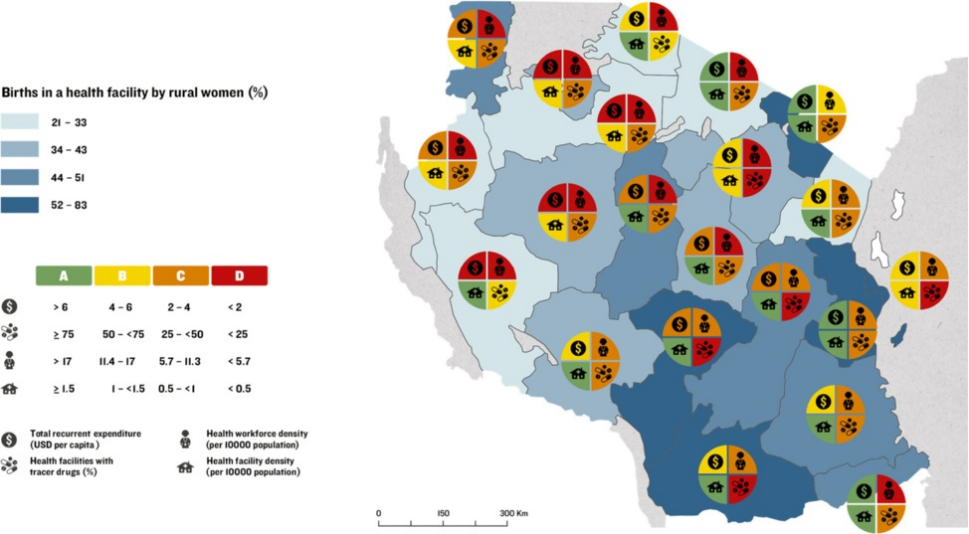
Map of Tanzania showing proportion of births by rural women in a health facility and barometers of regional health system implementation readiness

**Fig. 11:**
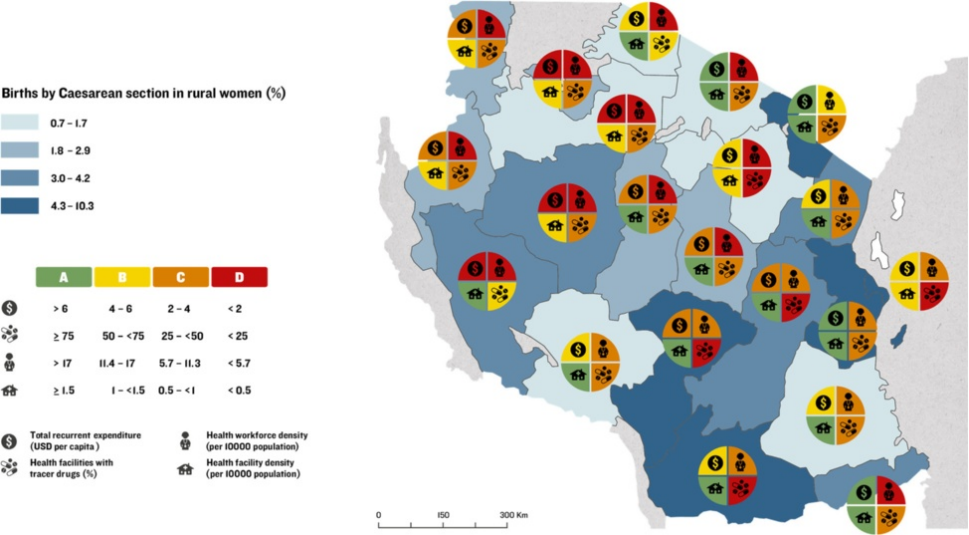
Map of Tanzania showing proportion of births by rural women by Caesarean section and barometers of regional health system implementation readiness

Data and grading for the implementation readiness barometers in each region are presented in Fig. 2. The barometers show mixed implementation readiness at regional level (Figs. 10 and 11). None of the regions meet the benchmarks for all four health systems building blocks, and North-eastern regions and Dar es Salaam have the weakest implementation readiness. Across Tanzania, health workforce and tracer drugs availability do not exceed 50 % of the required thresholds. There is a mixed picture of health system implementation readiness in relation to proportion of rural births in facilities and by C-section (Figs. 10 and 11). Both Dar es Salaam and neighbouring Pwani (to some extent urban environments) have higher proportions of rural facility births and C-sections, yet Dar es Salaam does not achieve a “green light,” for any single component of its barometer, whereas Pwani meets the threshold for health financing and facilities. Kilimanjaro performs well across health financing and health facility building blocks, and has highest density of skilled workforce, yet does not meet the benchmark for skilled workforce or drugs. Regions in the Lake (Kagera, Mara, Mwanza and Shinyanga) and Western Zones (Kigoma and Tabora) have the fewest “green light” building blocks and low coverage of care at birth services for rural women.

### Objective 4. Explore qualitatively the governance and management of health financial flows

Qualitative results revealed a bottom-up budget planning process (Additional file 1). Plans originate at the health facility governing committee at the council level (which include community members and health staff), and progress to the council health management team (CHMT) and district medical officers, and from there, to the regional and central level. The region approves budgets from each of its councils, and applies to central level for approval. In reality, the delayed receipt of funds are lower than the budget, resulting in the CHMT having to re-prioritise budgets and “*taking things out”* (district budget planner), i.e., no longer representing community priorities.

When asked about the process for prioritising different diseases areas, local government representatives reported that the facility managers were asked to provide a list of disease priorities, which members of the CHMT ranked using criteria provided by the central government. All the respondents identified that maternal health was their number one priority, driven by high-level support from the President and the development partners.

*“Malaria, HIV and maternal and newborn health are the top priorities, we will not get the budget approved if those three are not mentioned…”* (district budget planner)

Although this is positive given the clear need for investment in maternal health services, there was a lack of conformity at the local level about the decision to prioritise maternal health being a top-driven, vertical approach to maternal health, which prevented them from focusing on broader health systems issues that would have a direct impact on maternal health services, or other conditions they felt were a higher burden in their communities, such as malaria.

Respondents articulated that the central government department for drugs and commodities commonly experiences stock shortages, forcing local procurement of supplies through the private market, which was considered “*expensive*” a district government official, and therefore only used for “*very essential medicines*”. Substantial staff absenteeism was reported, due to (often donor-funded) training programmes.

A high degree of donor dependency was revealed at the district and regional levels, including for maternal health.

“*We [are] 100 % basket fund. If there’s no basket fund we cannot go to visit the facilities, for example. […] We can’t buy most of the supplies that are urgently needed and that currently may be frequently missing from medical stock.*” (CHMT member)

There was some disagreement on the level of donor influence on the budget process, with central-level respondents reporting stronger donor influence than subnational respondents. Nevertheless, respondents felt donors working on maternal health were generally better coordinated than those involved in other priorities.

## Discussion

This is the first study to map and analyse subnational health system readiness in for care at birth across each region in Tanzania, investigating finance, human and infrastructural resources, and visualising inequities with a novel implementation readiness barometer. This addresses the demand for greater disaggregation of country data [14, 17], and generates lessons for improved subnational implementation of care at birth services.

### Widespread subnational gaps in health system readiness for care at birth

We found considerable subnational variation across care at birth outcomes, and key health system and contextual indicators. All regions are critically under-resourced in workforce and essential commodities, although 14 of the 21 regions have an adequate density of health facilities. No region meets the benchmarks for all four health systems building blocks (health finance, health workforce, health facilities, and commodities). We highlight wide inequities in coverage of care between regions, both for rural births in facilities (21 to 90 %) and by C-section (1 to 10 %). We also found suboptimal availability and substantial inequity in commodities subnationally — essential tracer drugs were available at only 1 % of health centres and hospitals in Manyara region, while Rukwa, the best performing region, only reported availability of tracer drugs at 57 % of its health facilities.

The resulting maps with health system readiness barometers signpost clear improvements needed to improve care at birth outcomes. Despite the expectation that subnational data is often of weak quality and difficult to obtain, results agree with existing evidence [16, 17, 50] that measure MNH health system strength in Tanzania - demonstrating that analyses of available health system building block tracer indicators yield useful results for understanding health system readiness.

### Explaining variation in care at birth services for rural women

Health facilities, health workforce and quality of care have a strong and statistically significant association with coverage of care at birth for rural women in Tanzania. Specifically, significant positive associations were found between rural care at birth outcomes and maternal education, per capita recurrent government expenditure, health workforce density, health facility density and our measure of quality of care, while care at birth outcomes were negatively associated with proportion of births to rural women, TFR and stock-outs of essential commodities. Tanzania’s health financing system is revealed to be complex with insufficient disbursement of funds impeding service provision, and often dependent on external financing at the local level. However, just one association arose between financial indicators and rural care at birth (per capita recurrent expenditure and facility births) suggesting that additional modes of financing (donor funding, and CHF coverage) do not influence care at birth sub-nationally. CHF may be too low coverage overall to impact on care at birth. One key limitation of our financing analysis was that we found no data on sub-national basket fund disbursements. Qualitative data suggested the basket fund was crucial for health service provision at the district level, meaning we may have under-estimated the total effect of ODA (we rather represent the impact of vertically-delivered ODA).

We may see no association between financing and rural care at birth indicators because all financing levels are insufficient, resulting in no health system impact. Even when considering historical trends in outcomes, we still found no association with domestic investment, (for example, between the outcomes data from 2005 to 2010 and financial data from year 2012/13). Regions with the highest rate of fertility performed worst for health facility and human resourcing, quality of care, and both care at birth outcomes, indicating that high-burden regions are inadequately resourced according to need, and alluding that inadequate health services lead to higher fertility. Similarly, the lack of association between health readiness and care at birth may be a result of overall inadequate water supply at facilities, so no effect can be observed. This is supported by evidence in the literature that just 44 % of Tanzanian health facilities providing care at birth (and 24 % of delivery rooms) had adequate access to water and sanitation [39].

### Limitations and future research

Future research should utilise and promote disaggregate data from across the health system building blocks, and develop international guidance on appropriate inequity measurement in MNH [51, 52]. Access to and strengthening of existing routine data mechanisms are key to performing more robust analyses of health system strength for care at birth, and reducing inequities. An enabling environment, including access to necessary commodities and equipment, is essential to the provision of quality care at birth [53]. Yet accessing reliable subnational tracer indicator data was challenging and the commodities tracking system is reported to be weak [12]; the sources were not all well-matched by year (ranging from 2005 to 2014), which is problematic for interpreting true associations as there may be a lag in observing an effect; and rural disaggregation was not possible across all data. Subnational data was unavailable or not quantifiable for governance, health information systems, and legislative frameworks building blocks. Classification of rural context differed between sources, and classification of rural DHS data from highly urban context (e.g., Dar es Salaam) produces artefact errors. Some DHS data had low precision with wide 95 % confidence intervals due to weighted sampling (Additional file 1).

We used the same outcome indicators as the first Countdown case study for Tanzania [7] to allow us to build on a national average and present the inequities that underlie it, recognising the importance of C-section as a life-saving intervention was robust enough to justify its use as a proxy for emergency obstetric care. Yet this is likely an underestimation for all EmONC services, the most basic functions of which may be more available at health centre level.

Care at birth outcomes are assumed to be a good proxy for maternal and neonatal mortality [54], although additional subnational health system data is needed to explain variation in coverage. Reliable, publically accessible health utilisation data, readiness, and quality of care data – components of the service delivery building block – were lacking at country and subnational level, while our proxy indicators could not capture all dimensions of these complex aspects. Although water and sanitation encompasses a basic essential capacity for care at birth, it does not capture all components of readiness, likewise our proxy for quality of care. For example, the available physical infrastructure, supplies, management, and human resources with the knowledge, skills and capacity to deal with pregnancy and childbirth normal physiological, social and cultural processes, and life-saving interventions [55], as well as ignoring complex experiential factors such as mistreatment and disrespect (a widespread problem undermining the health care system in Tanzania) [56, 57]. We could not disaggregate health workforce data to staff with actual midwifery and obstetric care responsibilities, and could not restrict facility data to public only. We had to assume that health centres and hospitals could provide emergency obstetric care, although sources suggest that more than 50 % are not fully CEmOC compliant (16 % of hospitals and 89 % of health centres do not provide C-section deliveries) [38]. It remains problematic that health facility data sources do not disaggregate to facilities providing maternity and CEmOC services, at subnational level. CEmOC evaluations in certain regions, for example Kigoma [58], provide valuable, context-specific insights to the decentralisation of CEmOC services. Financial indicators were disaggregated inconsistently, meaning we compared funding streams for health (recurrent and OOP expenditure) and MNCH (ODA). Financial data were somewhat incomplete and we had to supplement with additional data. Domestic expenditure figures were mostly incomplete for development expenditure; we therefore selected recurrent expenditure as an indicator of government expenditure but this is an under-estimate of total government expenditure on health.

This signals the need for systematic subnational focus on data for quality of care, human resources, emergency obstetric care facilities and care at birth outcomes (rather than rely on five-yearly DHS surveys), more prolific subnational analysis of existing data [38, 59], and the need to make such data (including HMIS) publically available for analysis. Further exploration is needed to assess why certain regions perform better for care at birth coverage despite weak health system readiness.

Our study highlights both the current possibilities in subnational analysis and the gaps in data quality that must be addressed. Our analyses demonstrate that, despite expectations that subnational data are weak or inaccessible, analysing available health system building block tracer indicators yield useful results for decision-making. Nevertheless, improving subnational data quality and availability must remain a priority [17].

Our study is consistent with existing literature signalling the need for the development and validation of internationally agreed benchmarks for WHO health systems building blocks [46]. As there are no international standards for commodities and health financing, we used those developed for a previous study that have not yet been validated [46]. Benchmarks for health workforce and facilities vary widely – for example, Gabrysch et al. [45] recommend defining density of skilled health workforce by births denominator rather than population when assessing obstetric care-related outcomes, yet WHO’s minimum density threshold is 23 skilled health professionals per 10,000 population [34], and the International Labour Organisation recommendations vary from 35 [60], to 41 health workers per 10,000 population [61]. We used the most conservative WHO benchmark of 23 skilled health workforce per 10,000 population, yet even the best-performing region, Kilimanjaro, fell well below this at 12 skilled health workforce per 10,000 population. Hence our traffic light categorisation of data could be interpreted as reductive, as even the “green lighted” regions are not necessarily adequately financed - receiving just 6–8 USD per capita from the central government.

Although our theoretical framework stipulates each health system input as essential components, overall performance is driven by collective strength [18]. Future analyses should also incorporate data on governance and leadership, demand and community engagement, and efficiency in use of resources. Regions that improve service provision and overcome challenges should be explored and championed. Some answers may not lie in the health system, and multi-sectoral approaches to strengthening should be explored. Access to services may be shaped by distal determinants, given strong social and cultural factors, at the individual-, family- and community-level in Tanzania [62], and some rural areas such as Kagera benefit from robust road networks and military infrastructure [63].

## Conclusions

### National and global implications

Our results highlight wide inequities for Tanzania to urgently address in rural areas and where TFR is greatest, especially the low health system inputs of skilled health workers and essential RMNCH drugs and commodities, and insufficient health financing in the Lake and Western Zones (Table 2). Regions that perform well despite limited resources may hold some lessons for success.

**Table 2:** Key messages

***Key messages***
1. **Novel analysis of subnational variation in health systems inputs:** This is the first study to use GIS mapping techniques to visualise subnational health system readiness for care at birth across mainland Tanzania, and particularly to better understand this variation by examining finance and other inputs.
2. **Widespread subnational gaps in health system readiness for care at birth:** No region meets the benchmarks for all four health systems building blocks including for health finance, health workforce, health facilities, and commodities.
3. **Explaining variation in care at birth services for rural women:** Significant correlations were found between proportion of rural women delivering in a health facility, and by Caesarean section with health system readiness indicators including human resources density, health facility density, availability of essential commodities, and quality of care. However, some outlier Tanzanian regions, e.g., Kagera, demonstrate improved delivery of care at birth services for rural women despite receiving suboptimal resources; which has implications for similar settings, and needs further investigation to understand why and how this is occurring.
4. **Research agenda:** Future research to improve care at birth services for rural women should take into account data on governance and leadership, demand and community engagement, and efficiency in use of resources. Further investigation is needed into understanding positive outlier regions within Tanzania demonstrating improved service provision despite facing resourcing challenges. Considerable research is also needed to improve health systems input data, particularly on health workforce and standard benchmarks.

The very strong positive association between health workforce and both care at birth indicators (CC = between 0.69 and 0.75, both *p*-values <0.01) and the inadequate staffing levels across the country, highlight this priority area for urgent attention – in agreement with existing health system readiness literature in Tanzania [16, 17]. The relationship between care at birth and financing is less clear from our data, as only per capita recurrent expenditure was significantly associated. It is possible that the provision of care through many facilities, in such a low-resource setting, places unmanageable pressure on scant human resources. The difficulties faced by MNH health workers and managers within an unsupportive and bureaucratic system, and non-existent professional development opportunities, are well articulated [64]. Strong effective managers and supportive supervision may be a key factor in quality health care in resource-poor settings with overwhelmed staff [65, 66]. Emerging evidence from the Tanzanian context suggests that concentrating skilled staff in fewer front line facilities, with higher-volume case management, could improve care at birth, and cause minimal loss of population coverage in rural areas [67, 68]. As poor rural women bypass facilities (and cross administrative boundaries) with inadequate care at birth provision [50, 69, 70], such a solution may be most pragmatic where skilled health workers are in critical shortage. Other researchers found dysfunction in district level decision-making, with broken relationships between the CHMT and the Council health services board [71].

With the SDGs prioritising Universal Health Coverage as a social protection mechanism [72] to counter inequity in health, multiple disaggregated indicators will soon be tracked, aiming to decrease disparities within countries and ensure that no-one is left behind. High-burden countries need to capitalise on opportunities to venture further in unmasking subnational inequities and outliers where lessons can be learnt and shared [73–75]. Government-led strengthening of HMIS systems in tandem will be essential for data quality improvement and availability. The World Bank’s Global Financing Facility (GFF) for RMNCH should capitalise on the subnational analytical approach to direct resourcing in a highly targeted manner, and to exploit the GFF’s access to government data to improve its quality and availability.

## Abbreviations

ANC, antenatal care; CEmOC, comprehensive emergency obstetric care; CHF, Community Health Fund; CHMT, Council Health Management Team; EmONC, emergency obstetric and newborn care; GDP, gross domestic product; GFF, Global Financing Facility; GIS, geographical information system; HMIS, Health Management Information System; HSP, health systems and policies; MDG, Millennium Development Goal; MNCH, maternal newborn and child health; MNH, maternal newborn health; MoHSW, Ministry of Health and Social Welfare; NHIF, National Health Insurance Fund; ODA, Official Development Assistance; OOP, out of pocket expenditure; PMO-RALG, Prime Minister’s Office for Regional Administration and Local Government; QOC, quality of care; RHMT, Regional Health Management Team; RMNCH, reproductive, maternal, newborn and child health; SDG, Sustainable Development Goal; TFR, total fertility rate; TZS, Tanzanian shillings; USAID, United States Agency for International Aid; USD, United States Dollar; WHO, World Health Organisation

## Additional file

Additional file 1: Supplementary appendix. (DOCX 13018 kb)
