# Supplementary material for: Subnational variation for care at birth in Tanzania: is this explained by place, people, money or drugs?
Source: BMC Public Health. 2016 Sep 12;16(Suppl 2):795. doi: 10.1186/s12889-016-3404-3 (PMC5025821; doi:10.1186/s12889-016-3404-3)
Supplement: Additional file 1: — Supplementary appendix. (DOCX 13018 kb) [file 12889_2016_3404_MOESM1_ESM.docx]

**Subnational variation for care at birth in Tanzania: is this explained by place, people, money or drugs?**

**Additional file 1**

Contents

[A. Definitions 2](#_Toc450635567)

[B. Data 3](#_Toc450635568)

[i. Table 1. Contextual data 3](#_Toc450635569)

[ii. Table 2. Table of inputs, outputs and outcomes data 4](#_Toc450635570)

[iii. Table 3 List of Tracer Medicines in Tanzania 5](#_Toc450635571)

[iv. Births demographics data 6](#_Toc450635572)

[v. Health service readiness data 6](#_Toc450635573)

[vi. Financial quantitative and qualitative data 6](#_Toc450635574)

[vii. Table 4. List of Partners Consulted for the Report on Reproductive, Maternal, Newborn and Child Health Partners Mapping and Resource Tracking, Budget FY 2013-2014. 7](#_Toc450635575)

[viii. DHS analysis 7](#_Toc450635576)

[ix. Regional Boundary re-coding 7](#_Toc450635577)

[x. Additional maps 9](#_Toc450635578)

[xi. Correlations 14](#_Toc450635579)

C. References

## Definitions

ANC Antenatal Care

CHF Community Health Fund

DHS Demographic Health Surveys

GDP Gross Domestic Product

HMIS Health Management Information System

MDG Millennium Development Goal

MoHSW Ministry of Health and Social Welfare

MNH Maternal and Newborn Health

MNCH Maternal, Newborn and Child Health

NHA National Health Account

NIHF National Health Insurance Fund

ODA Official Development Assistance

OECD-CRS Organisation for Economic Co-Operation and Development’s Creditor Rating System

OOP Out of Pocket Expenditure

PMO-RALG Prime Minister’s Office for Regional Administration and Local Government

QoC Quality of Care

RHMT Regional Health Management Team

RMNCH Reproductive, Maternal, Newborn and Child Health

TFR Total Fertility Rate

USAID United States Agency for International Aid

UN United Nations

USD United States Dollar

WHO World Health Organization

## Data

### Table 1. Contextual data

###

### Table 2. Table of inputs, outputs and outcomes data

*No CHF data for Dar es Salaam

**Water is piped into the facility or piped onto facility grounds, or else water comes from a public tap or standpipe, a tube well or a borehole, a protected dug well, protected spring, or rain water or bottled water, and the outlet from this source is within 500 metres of the facility.

### Table 3. List of Essential Tracer Medicines in Tanzania

|  | **Medicine/Supply** | **Service Category** |
| --- | --- | --- |
| 1. | DPT + HepB/ HiB vaccine | Immunisation/Child |
| 2 | ACT for Malaria | Malaria (Child and Women and general Population) |
| 3. | Amoxycillin and Cotrimoxazole Syrup | Child |
| 4. | Amoxycillin/ Cotrimoxazole Caps/Tabs | Children/Adults |
| 5. | Albendazole/Mebendazole Tabs | Children/Adults |
| 6. | ORS for treatment of Diarrhoeas | Children/Adults Management of Diarrhoea |
| 7. | Tabs Zinc Sulphate for Treatment of Diarrhoea | Children/management of Diarrhoea |
| 8. | Injection Oxytocin and Ergometrine au Oxytocin and/or Misoprostol | Childbirth management |
| 9. | Injection Magnessium Sulphate | Preeclampsia/Eclampsia management |
| 10. | Injection Depoprovera for family Planning | Family Planning |
| 11. | Combine Oral Contraceptives | Family Planning |
| 12. | Ferrous + Folic Acid Tablets | Management of Anaemia especially in pregnancy |
| 13. | mRDT for malaria | Malaria Diagnosis |
| 14. | Infusions (Dextrose 5% and/or Dextrose Saline) |  |
| 15. | Syringes (Disposable |  |
| 16. | Tabs Paracetamol | Management of pain, fever |
| 17 | Injection Benzyl Penicillin | Infection management |
| 18 | Metronidazole Tablets | Infection management |
| 19 | Nevirapine Oral solution | ART for children |
| 20 | Tenofovir 300mg+Lamivudine 300mg+ Efavirenz 600mg Tablets | ART (both PMTCT and HIV) |
| 21 | Efavirenz 600mg Tablet |  |
| 22 | Zidovudine 60mg+Lamivudine 30mg+Nevirapine 50mg Tablets |  |
| 23 | UNIGOLD HIV 1⁄2 |  |
| 24 | Determine HIV 1&2 |  |
| 25 | FACS Count reagent |  |
| 26 | DBS for Early Infant Diagnosis |  |
| 27 | RHZE Rifampicin 150mg/Isoniazide 75mg/Pyrazinamide /Ethambutol Tablets |  |
| 28 | RH Rifampicin 150MG/Isoniazide 75mg Tablets |  |
| 29 | Catgut Sutures |  |
| 30 | Sulphadoxine+pyrimethamine (SP) tablets | Management of Malaria in Pregnancy |

###

### Births demographics data

Population distributions were generated through the WorldPop project (available at worldpop.org.uk) incorporating satellite imagery data such as settlements, land cover, and night-time lights to redistribute census counts, deriving population distributions and projections of women of childbearing age on a 100m scale for the years 2010-2035. Urban and rural extents were defined using Global Rural-Urban Mapping Project (GRUMP) data, available through Columbia University(1). Sub-national urban and rural age-specific fertility rates were generated using 2010 Demographic and Health Survey (DHS) data(2) with a STATA program developed by Pullum(3), while UN population and births projections and other data sources(4) representing subnational age structure, fertility and growth rates were then incorporated into these population distributions to generate estimates of live births and pregnancies in Tanzania.(4) Finally, births data were adjusted to match annual national-level UN estimates of births per year, following medium fertility projections. For these analyses, mean birth density is represented on the 1 km scale, by region. Detailed methodology is outlined further in Tatem et al. 2014.(4) The national aggregate mean birth density was calculated using World Bank data for the total land mass of Tanzania(5), while the rural land mass was derived by subtracting the Worldpop satellite derived urban extents data (3366 km^2^)from the total land mass (885800 km^2^). One limitation is that the urban extents correspond to 1995, since when urban areas are likely to have grown in size.

### Health service readiness data

The proxy indicator for health service readiness is the percentage of all health facilities with an improved water source. This is defined as where water is piped into the facility or piped onto facility grounds, or else water comes from a public tap or standpipe, a tube well or a borehole, a protected dug well, protected spring, or rain water or bottled water, and the outlet from this source is within 500 metres of the facility.

### Financial quantitative and qualitative data

Methods

We cross-referenced the ten main donors with the Countdown database, which collates project-level information on ODA for RMNCH by country and donor(6) to assess consistency in reporting. The Ministry of Health and Social Welfare of Tanzania’s Report on Reproductive, Maternal, Newborn and Child Health Partners Mapping and Resource Tracking, Budget FY 2013-2014 lacked Official Development Assistance data from United States Agency for International Development (USAID) (Webappendix table 3). Thus the authors contacted the Tanzania USAID country office and through personal communication received the breakdown of USAID-funded RMNCH projects to MNH for year 2013-14. Project funding was reported in total number of US dollars with a description of the type of support provided, eg Maternal and Neonatal Health, Child Health, and whether provided at National or Regional level. USAID-supported regions were Arusha, Dodoma, Kigoma, Iringa, Morogoro, Shinyanga, Singda and Tabora. Where a project spanned multiple regions the total funding was assumed to be divided equally among the regions.

Tanzania’s health budget is allocated according to a resource formula that accounts for population, under-five mortality and poverty levels(7). However, the only data on these were from 2002(7), and thus deemed too old to be meaningful in this analysis. In addition, the formula is 70% weighted on population, therefore we feel the figures used are adequate estimates of the subnational distribution of resources.

Results

External resources (ODA) are allocated either to the Ministry of Finance in the form of general budget support, to the MoHSW in the form of the ‘health basket fund’ or vertical projects, and to the regional and council health management teams in the form of vertical projects and the basket fund. Domestic tax-based funding is channelled through the Ministry of Finance to the Prime Minister’s Office for Regional Administration and Local Government (PMO-RALG) and MoHSW at the central level, to the Regional Health Management Team (RHMT) and to the Council Health Management Team (CHMT) in the form of the health block grant. Councils raise funds from OOP from individuals, the CHF, the drug revolving fund and the councils’ own sources.

### Table 4. List of Partners Consulted for the Report on Reproductive, Maternal, Newborn and Child Health Partners Mapping and Resource Tracking, Budget Financial Year 2013-2014.

| # | Name | Type |
| --- | --- | --- |
| 1 | AGOTA | Development Partner |
| 2 | AKHST | Development Partner |
| 3 | AMREF | Development Partner |
| 4 | Baylor, Concern, CUAMM, Private Sector, TFNC | Development Partner |
| 5 | CARE | Development Partner |
| 6 | CCBRT | Development Partner |
| 7 | CHAI | Development Partner |
| 8 | CSO | Other |
| 9 | EAC | Development Partner |
| 10 | EngenderHealth/ MoHSW/ UNFPA | Development Partner |
| 11 | Evidence for Action (E4A) Tanzania | Development Partner |
| 12 | FHI360 | Development Partner |
| 13 | Government of Tanzania | Government |
| 14 | Haydom Lutheran Hospital | Development Partner |
| 15 | ICAP | Development Partner |
| 16 | KfW | Development Partner |
| 17 | KIWOHEDE/UNFPA | Development Partner |
| 18 | LGA | Government |
| 19 | M2M | Development Partner |
| 20 | Management and Development for Health | Development Partner |
| 21 | Medicos del Mundo | Development Partner |
| 22 | MoHSW | Government |
| 23 | NHIF | Government |
| 24 | Norwegian Embassy | Development Partner |
| 25 | Plan International | Development Partner |
| 26 | PMO | Development Partner |
| 27 | PRINMAT | Development Partner |
| 28 | PSI | Development Partner |
| 29 | Save the Children International | Development Partner |
| 30 | Tanzania Health Promotion Support (THPS) | Government |
| 31 | TFNC | Government |
| 32 | UNFPA | Development Partner |
| 33 | UNICEF | Development Partner |
| 34 | Voluntary Service Overseas (VSO) | Development Partner |
| 35 | World Lung Foundation | Development Partner |
| 36 | World Health Organization | Development Partner |
| 37 | World Vision | Development Partner |

### DHS analysis

Data for all live births occurring within the five years preceding the 2010 DHS(2) were used, and are referred to in this analysis as a “birth”. For multiple pregnancies, the last child delivered was included in this analysis. DHS collects information on mode of delivery as a binary variable (vaginal or caesarean), thus births occurring at home or in a dispensary reported as either delivering by caesarean (7 births) or missing for mode of delivery (11 births) were recoded as a vaginal delivery.

The number of births by caesarean section in some regions were very low, resulting in wide confidence intervals.

### Regional Boundary re-coding

Our outcomes and QoC indicators used 2010 DHS data of births that happened between 2005 and 2010. Regional boundaries were re-calibrated in the years since the DHS in order to demarcate new districts, therefore any more recent data had to be disaggregated to district level and re-allocated/approximated to the 2010 regions (Figures A and B). In 2012 four regions were created. Geita was formed of one of Kagera districts, four from Mwanza and one from Shinyanga, thus we incorporated Geita data into Mwanza. Njombe was formed of Iringa, thus we classified Njombe data as Irigina. Simiyu was formed of Shinyanga, thus we merged these data to Shinyanga. Katavi was formed of Rukwa and thus these data were classified as Rukwa.

**Figure A**

**Map of Tanzania – Regional boundaries from years 2003 to 2011**

**Figure B**

**Map of Tanzania – Regional boundaries from years 2012 to present**

### Additional maps

Figure C – Health facilities providing care at birth vs. rural birth density in Tanzania


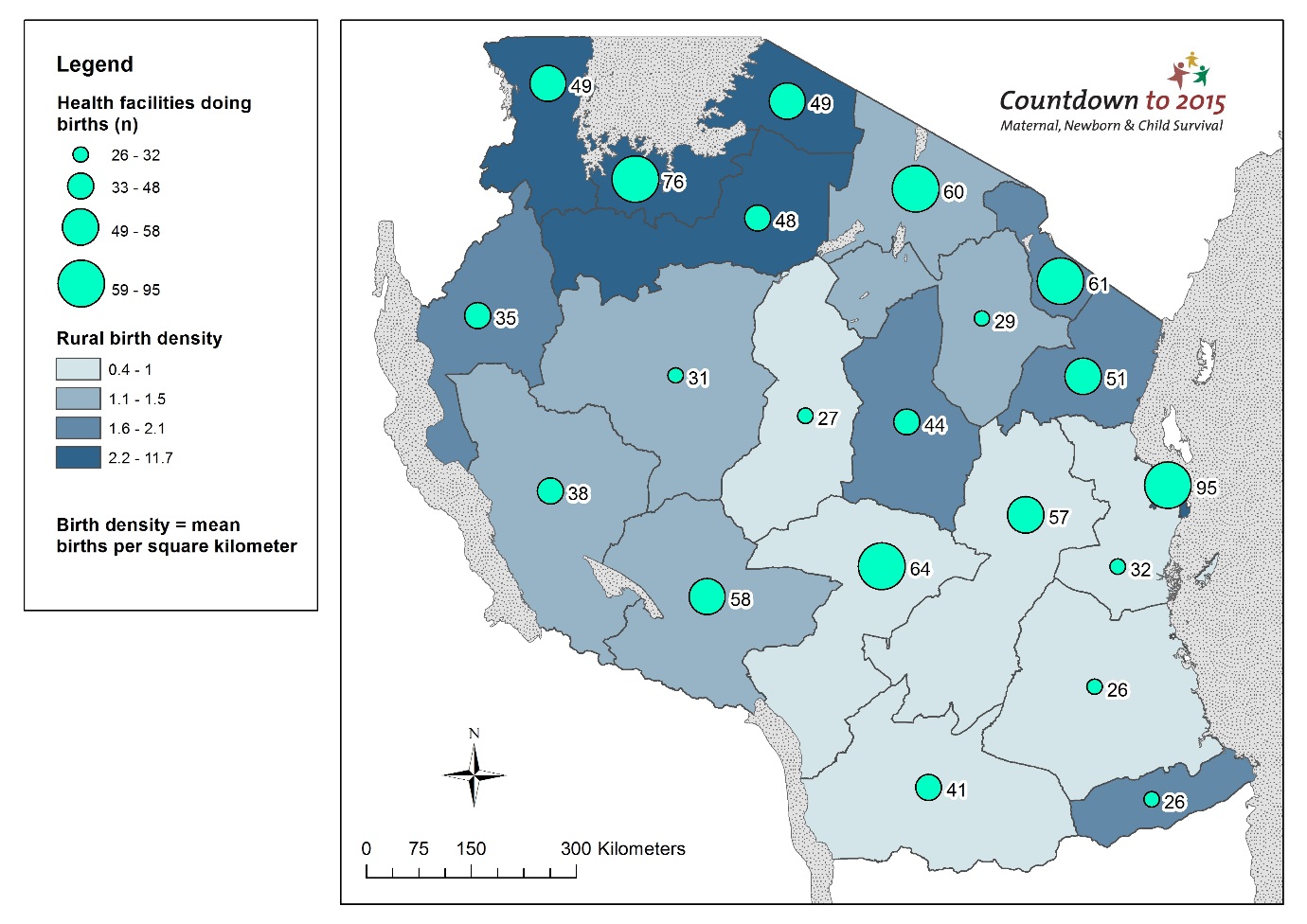


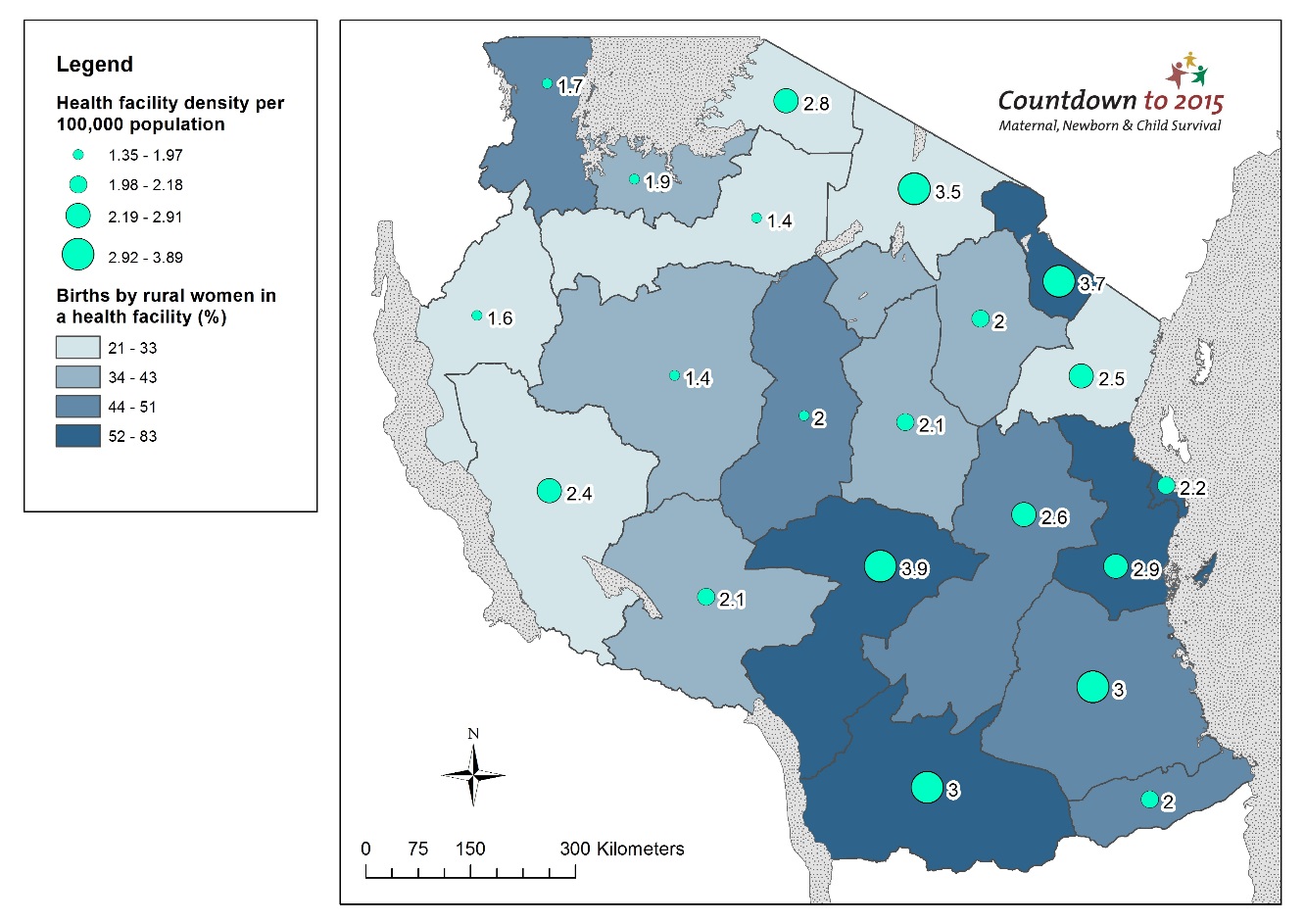


Figure D – Health facility density vs. births by rural women in a health facility in Tanzania


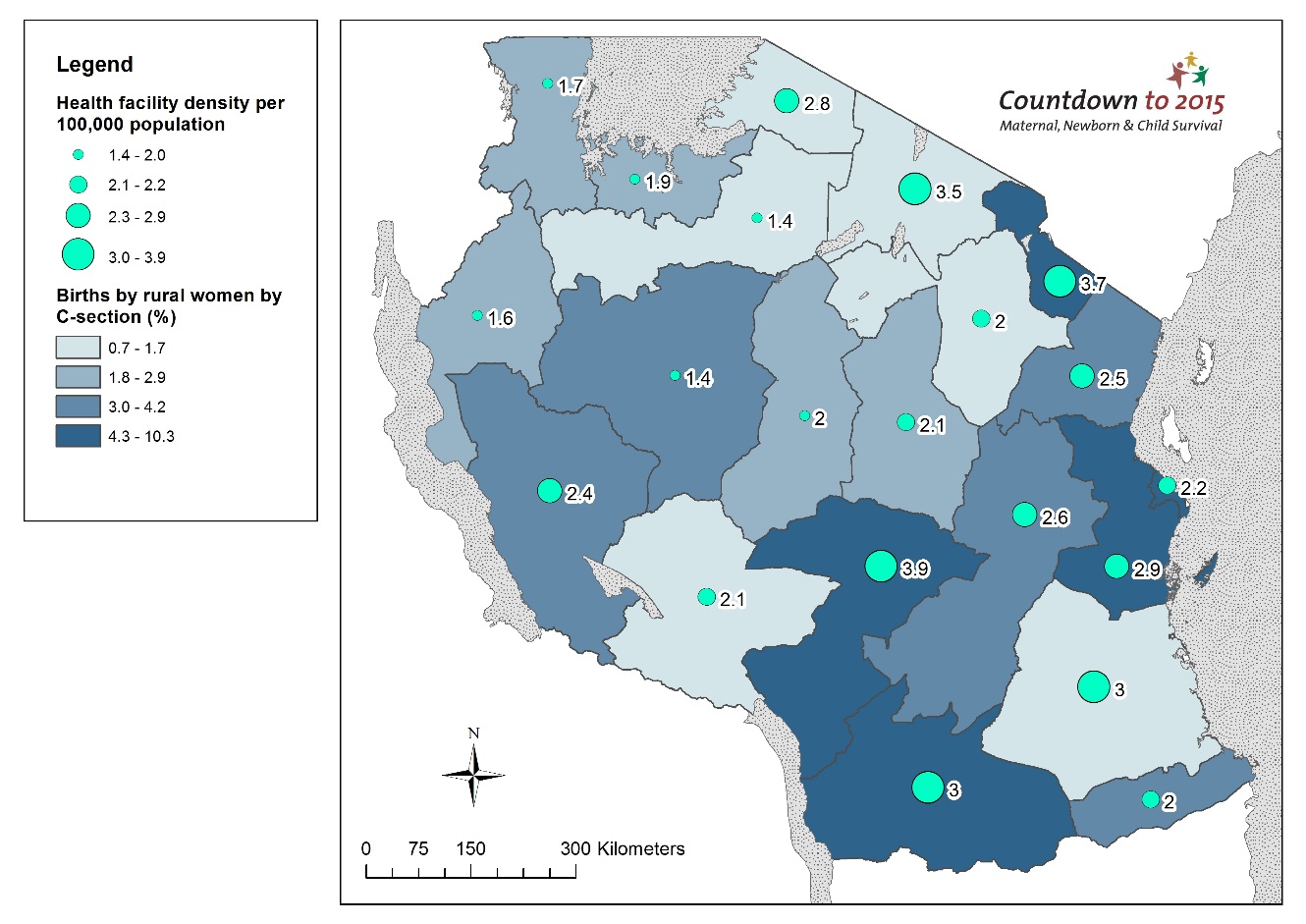


Figure E – Health facility density vs. births by rural women by C-section in Tanzania

The following maps present the outcome indicators of births by rural women (i) in a facility and (ii) by c-section at regional level, using denominators recommended in Gabrysch *et al.* (2012)(8), i.e. density of doctors and midwives per 3600 births, and density of health facilities per 20000 births.


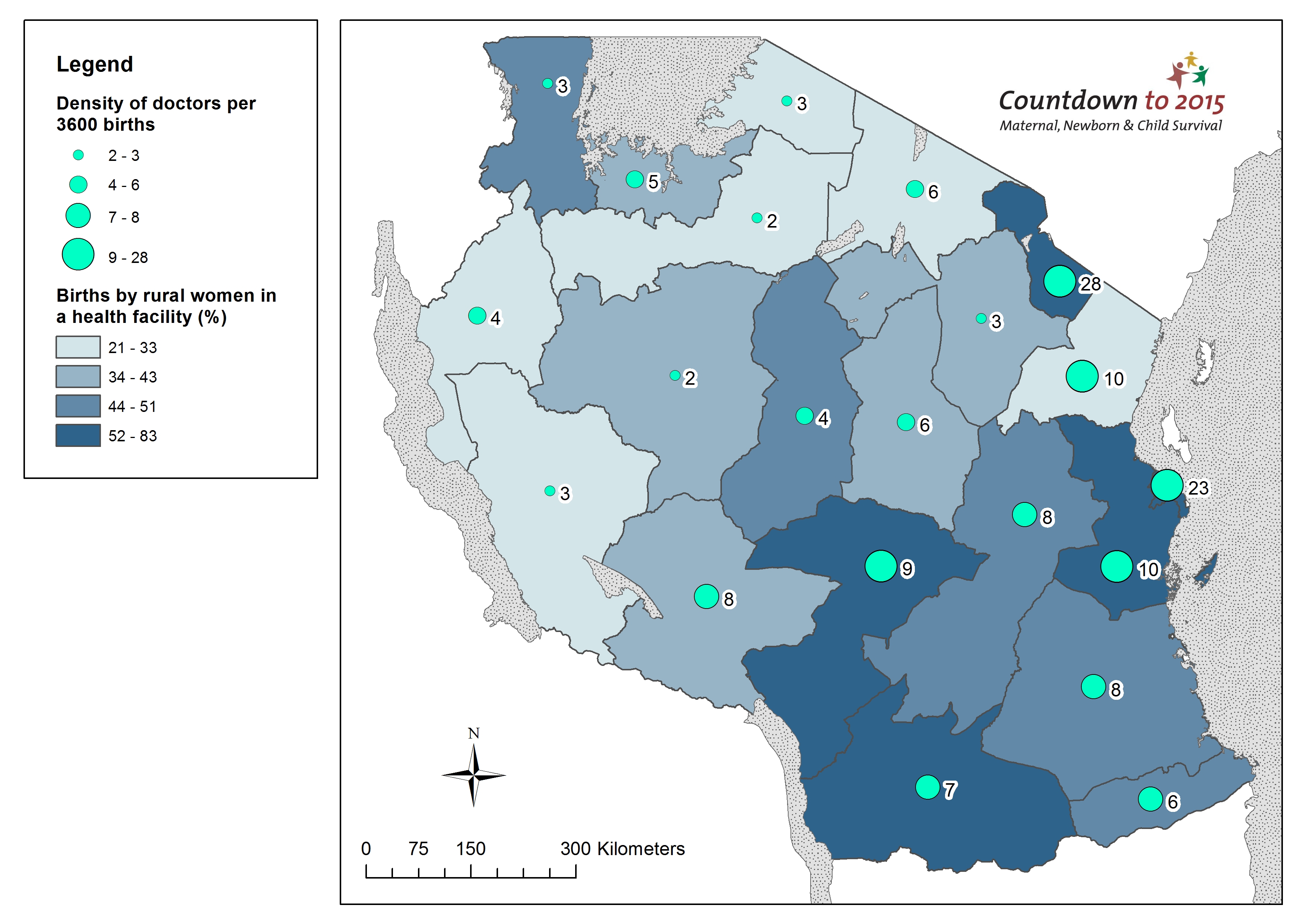


Figure F – Doctor density vs births by rural women in a health facility in Tanzania


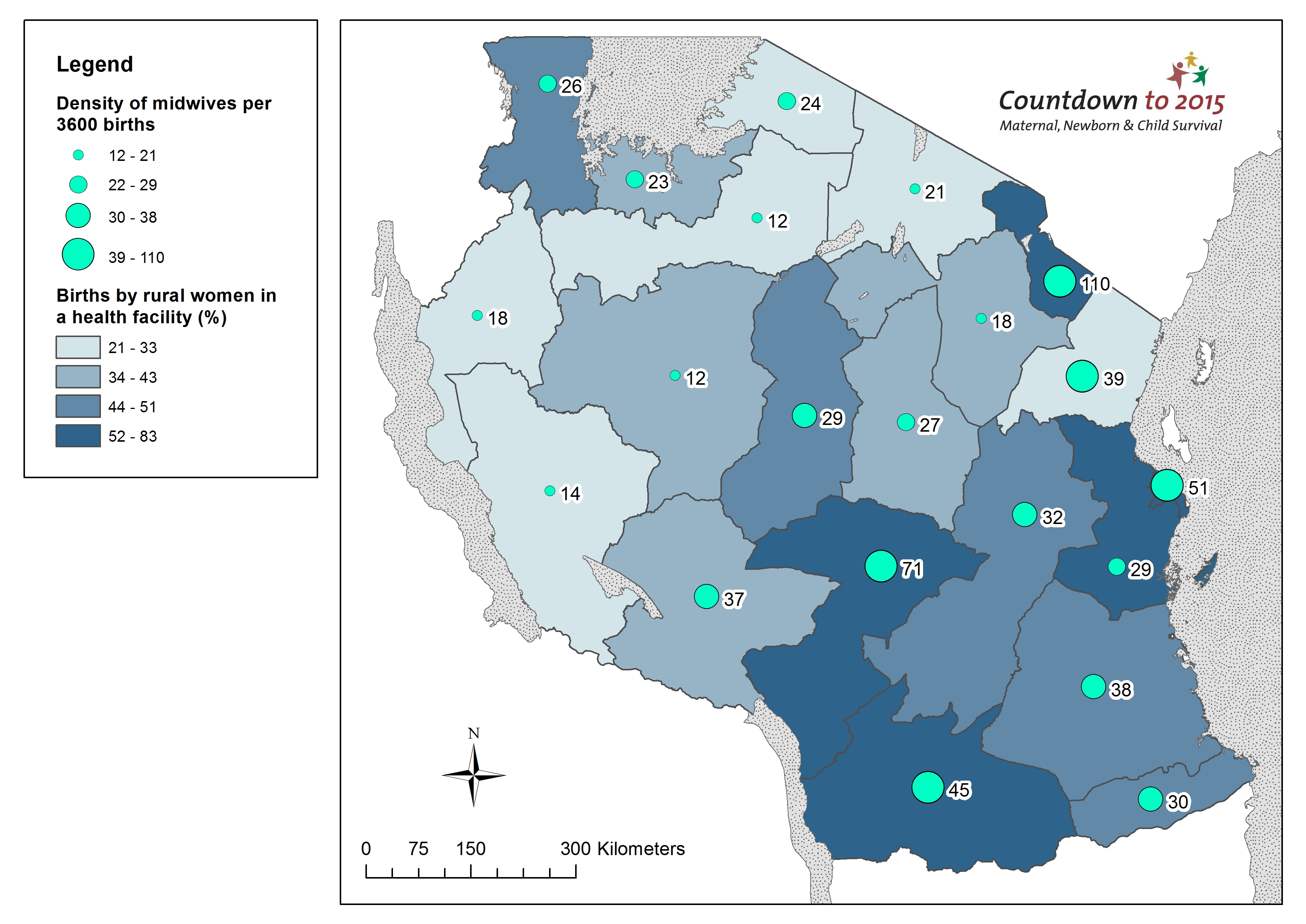


Figure G –Midwifery density vs births by rural women in a health facility in Tanzania

Figure H – Health facility density vs. births by rural women in a health facility in Tanzania


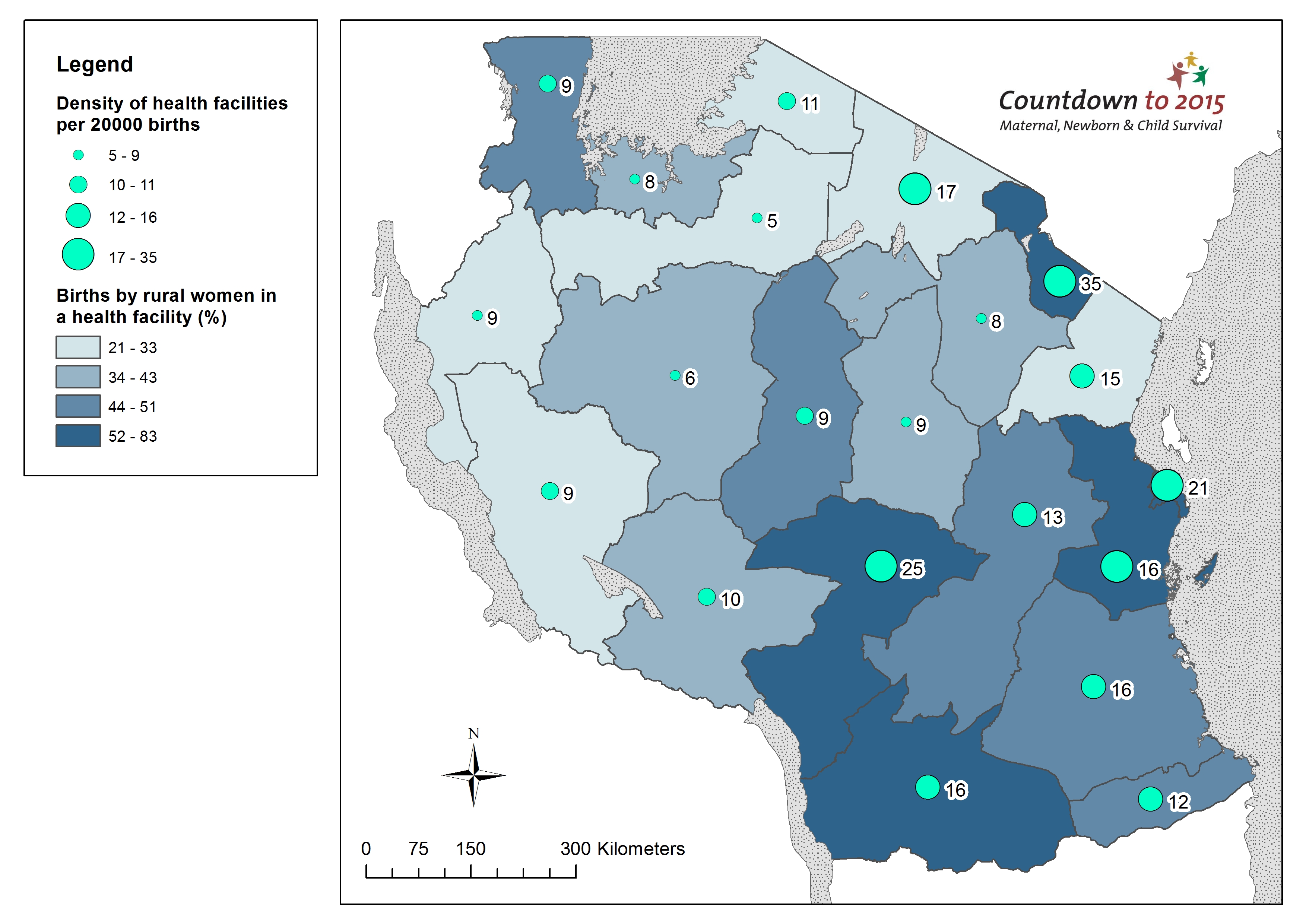


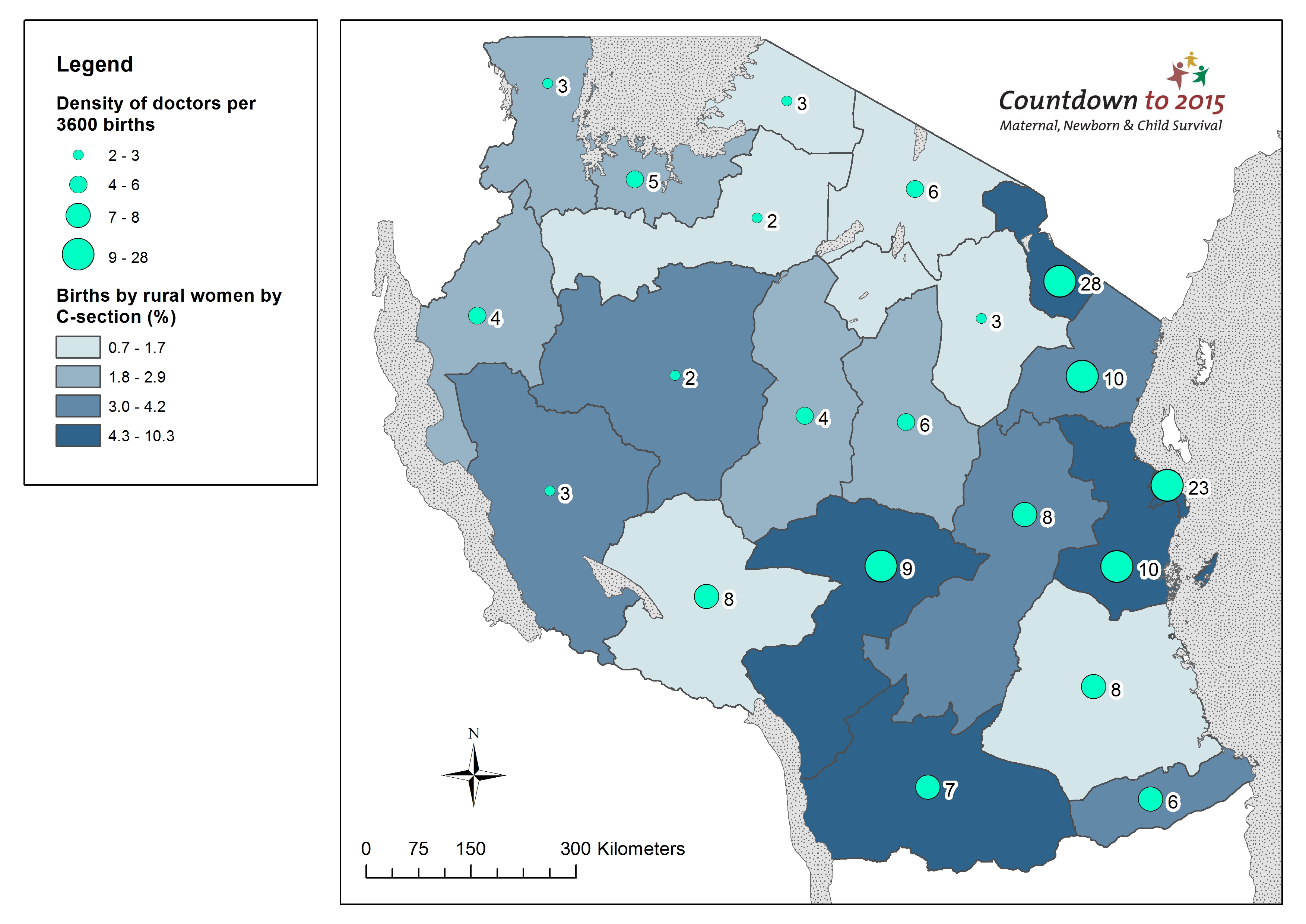


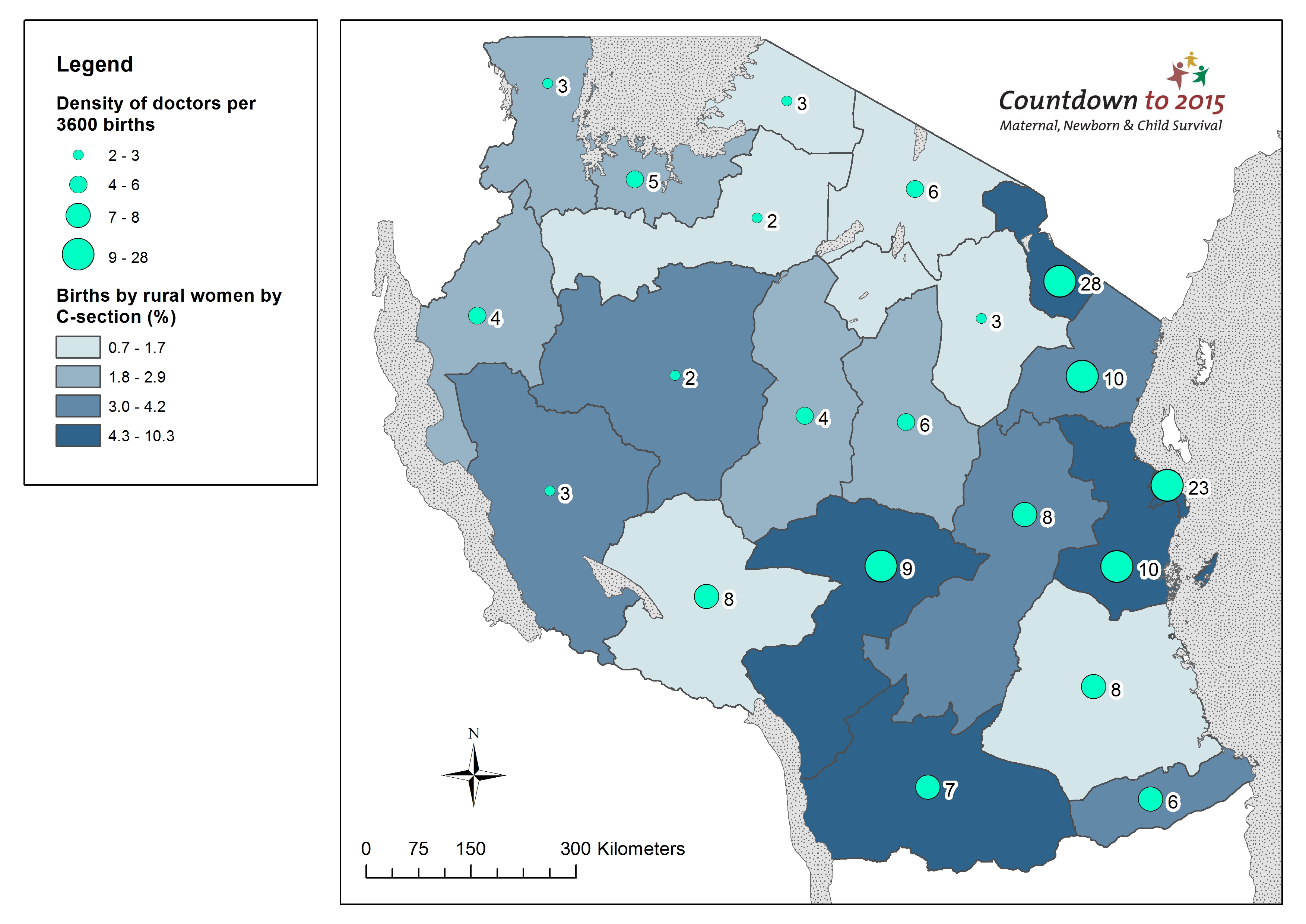


Figure I – Doctor facility density vs. births by rural women by C-section in Tanzania

Figure J – Midwifery density vs. births by rural women by C-section in Tanzania


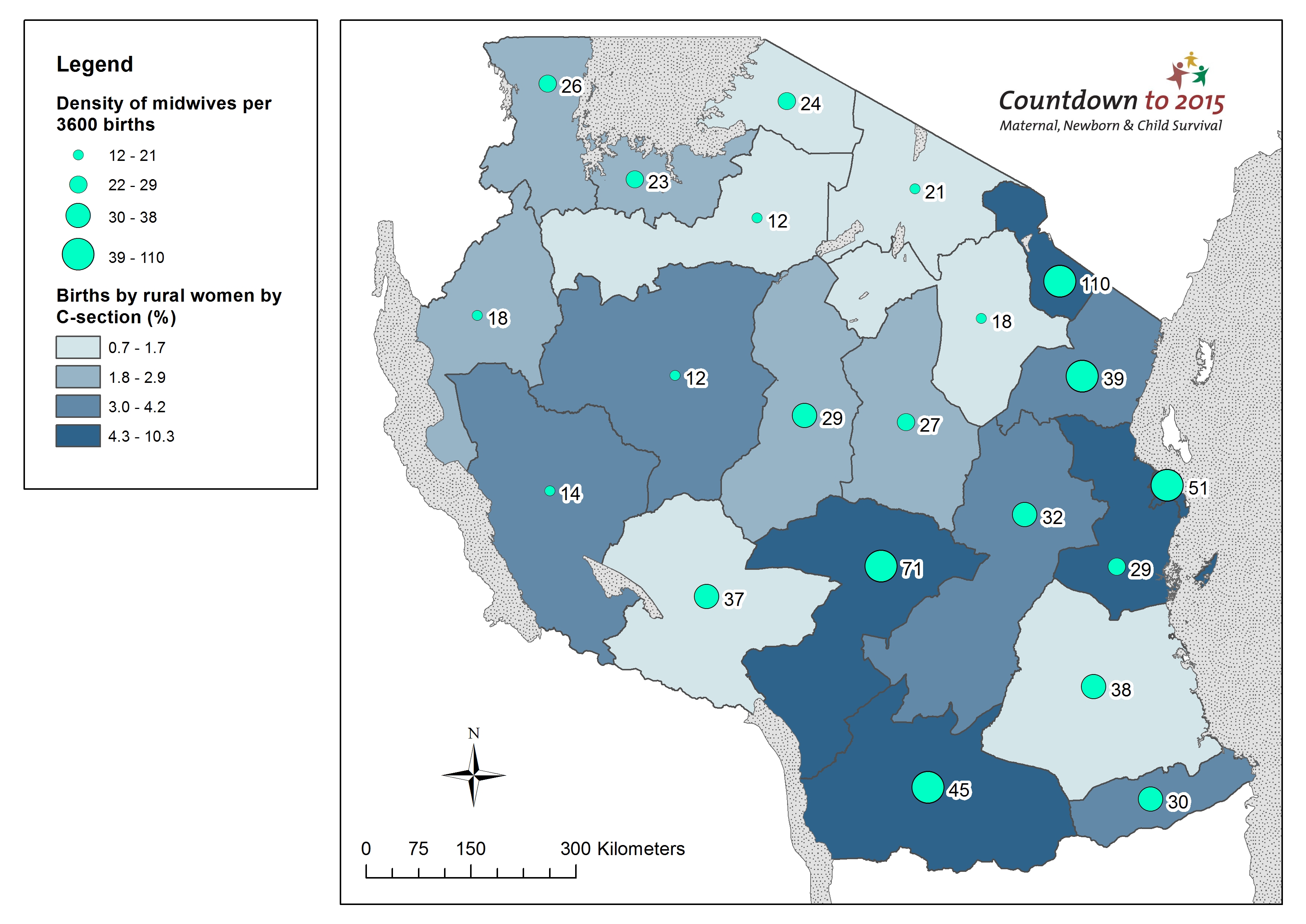


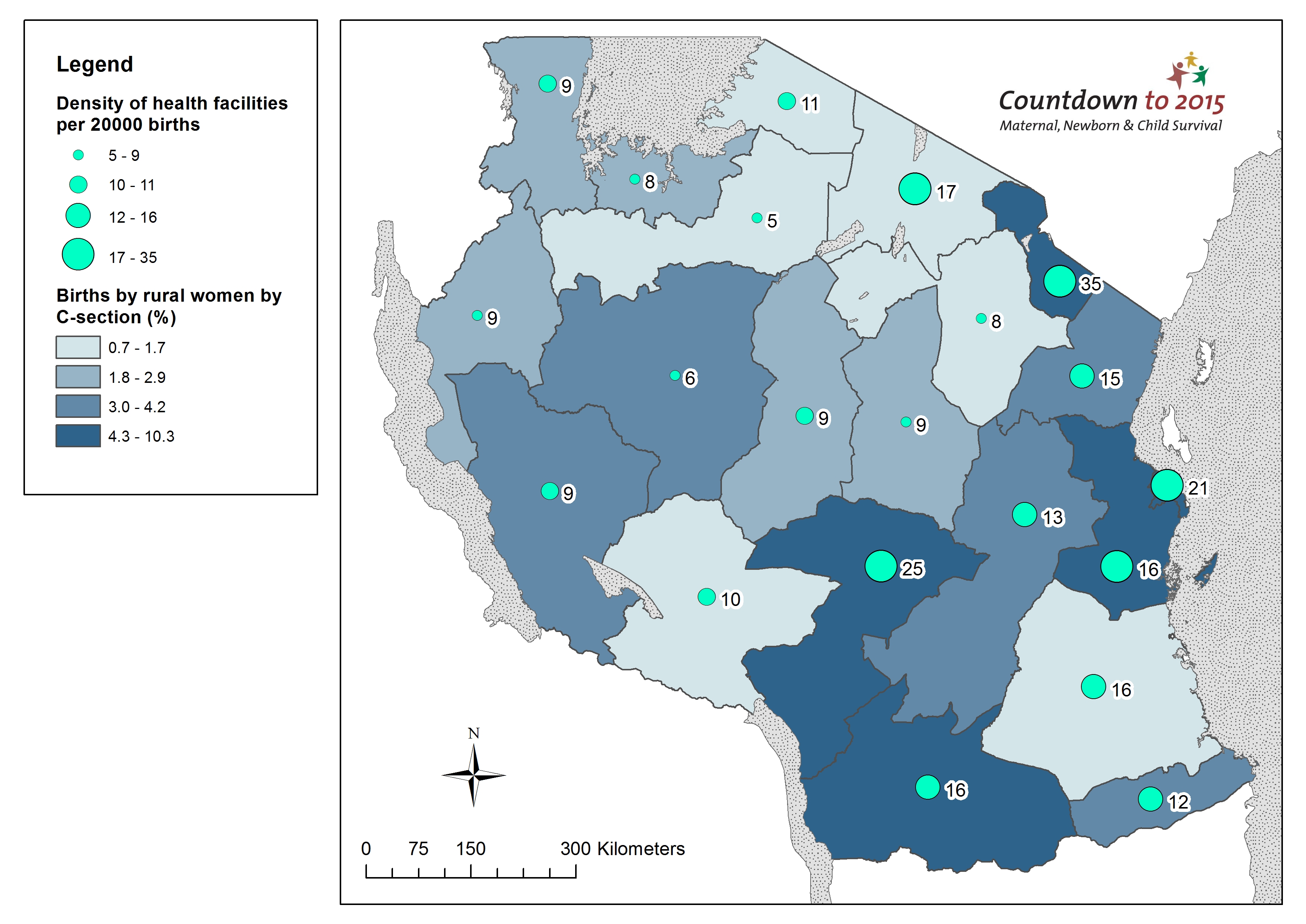


Figure K – Health facility density vs. births by rural women by C-section in Tanzania

### Correlations

| **Table 5) Correlation analysis between input and output indicators** | | | |
| --- | --- | --- | --- |
| **Input indicators** | **Co-efficient & P-value** | **Output indicator** | |
|  |  | **Women informed of signs of pregnancy complications at ANC** | **Facilities with improved water source** |
| Per capita recurrent health expenditure | Correlation co-efficient | 0.21 | 0.22 |
|  | P-value | 0.36 | 0.34 |
| OOP health expenditure | Correlation co-efficient | 0.20 | 0.27 |
|  | P-value | 0.40 | 0.23 |
| Community health fund coverage | Correlation co-efficient | 0.16 | 0.01 |
|  | P-value | 0.49 | 0.96 |
| ODA per capita to RMNCH | Correlation co-efficient | 0.01 | -0.22 |
|  | P-value | 0.96 | 0.34 |
| Skilled health workforce density by population | Correlation co-efficient | 0.44 | 0.12 |
|  | P-value | 0.04 | 0.59 |
| Skilled health workforce density by births | Correlation co-efficient | 0.55 | 0.30 |
|  | P-value | 0.01 | 0.18 |
| Health facility density by population | Correlation co-efficient | 0.28 | 0.08 |
|  | P-value | 0.22 | 0.74 |
| Health facility density by births | Correlation co-efficient | 0.56 | 0.29 |
|  | P-value | 0.01 | 0.20 |
| % Health facilities with tracer drugs | Correlation co-efficient | -0.32 | -0.22 |
|  | P-value | 0.15 | 0.33 |

| **Table 6) Correlation analysis between input and outcome indicators** | | | |
| --- | --- | --- | --- |
| **Input indicators** | **Co-efficient & P-value** | **Outcome indicators** | |
|  |  | **Proportion of births by rural women in a health facility** | **Proportion of births by rural women by C-Section** |
| Per capita recurrent health expenditure | Correlation co-efficient | 0.43 | 0.33 |
|  | P-value | 0.05 | 0.14 |
| OOP health expenditure | Correlation co-efficient | 0.21 | 0.27 |
|  | P-value | 0.35 | 0.24 |
| Community health fund coverage | Correlation co-efficient | 0.00 | -0.01 |
|  | P-value | 0.99 | 0.98 |
| ODA per capita to RMNCH | Correlation co-efficient | -0.01 | -0.14 |
|  | P-value | 0.95 | 0.56 |
| Skilled health workforce density by population | Correlation co-efficient | 0.73 | 0.69 |
|  | P-value | <0.01 (0.0002) | <0.01 (0.0005) |
| Skilled health workforce density by births | Correlation co-efficient | 0.71 | 0.75 |
|  | P-value | < 0.01 (0.0003) | <0.01 (0.0001) |
| Health facility density by population | Correlation co-efficient | 0.52 | 0.58 |
|  | P-value | 0.02 | 0.01 |
| Health facility density by births | Correlation co-efficient | 0.73 | 0.81 |
|  | P-value | <0.01 (0.0002) | <0.01 (0.0000) |
| % Health facilities with tracer drugs | Correlation co-efficient | -0.45 | -0.31 |
|  | P-value | 0.04 | 0.16 |

| **Table 7) Correlation analysis between contextual and input indicators** | | | | | | | | | | |
| --- | --- | --- | --- | --- | --- | --- | --- | --- | --- | --- |
| **Contextual indicator** | **Co-efficient & P-value** | **Input indicators** | | | | | | | | |
|  |  | **Per capita recurrent health expenditure** | **OOP health expenditure** | **Community health fund coverage** | **ODA per capita to RMNCH** | **Skilled health workforce density by population** | **Skilled health workforce density by births** | **Health facility density by population** | **Health facility density by births** | **% Health facilities with tracer drugs** |
| Total population | Correlation co-efficient | -0.49 | 0.53 | -0.09 | -0.23 | -0.05 | 0.08 | -0.47 | -0.17 | -0.08 |
|  | P-value | 0.02 | 0.01 | 0.70 | 0.31 | 0.83 | 0.74 | 0.03 | 0.45 | 0.71 |
| GDP per capita | Correlation co-efficient | 0.26 | 0.62 | -0.32 | -0.09 | 0.61 | 0.60 | 0.54 | 0.62 | -0.34 |
|  | P-value | 0.25 | <0.01 (0.0027) | 0.17 | 0.69 | < 0.01 (0.0034) | <0.01 (0.0038) | 0.01 | <0.01 (0.0027) | 0.13 |
| Maternal education | Correlation co-efficient | 0.49 | 0.56 | 0.09 | -0.32 | 0.72 | 0.72 | 0.57 | 0.73 | -0.37 |
|  | P-value | 0.02 | 0.01 | 0.72 | 0.15 | < 0.01 (0.0003) | <0.01 (0.0002) | <0.01 (0.007) | <0.01 (0.0002) | 0.10 |
| Proportion of births by rural women | Correlation co-efficient | -0.10 | -0.63 | 0.14 | 0.18 | -0.34 | -0.50 | 0.13 | -0.24 | 0.31 |
|  | P-value | 0.66 | <0.01 (0.0022) | 0.56 | 0.43 | 0.14 | 0.02 | 0.58 | 0.30 | 0.17 |
| Rural Birth density | Correlation co-efficient | -0.17 | 0.60 | -0.13 | -0.27 | 0.12 | 0.29 | -0.25 | 0.07 | -0.18 |
|  | P-value | 0.47 | <0.01 (0.004) | 0.58 | 0.24 | 0.59 | 0.20 | 0.27 | 0.77 | 0.43 |
| TFR | Correlation co-efficient | -0.64 | -0.38 | 0.08 | 0.15 | -0.74 | -0.79 | -0.54 | -0.81 | 0.49 |
|  | P-value | <0.01 (0.002) | 0.09 | 0.75 | 0.51 | <0.01 (0.0001) | <0.01 (0.0000) | 0.01 | <0.01 (0.0000) | 0.02 |

| **Table 8) Correlation analysis between contextual and output indicators** | | | |
| --- | --- | --- | --- |
| **Contextual indicators** | **Co-efficient & P-value** | **Output indicators** | |
|  |  | **Women informed of signs of pregnancy complications at ANC** | **Facilities with improved water source** |
| Total population | Correlation co-efficient | 0.07 | -0.13 |
|  | P-value | 0.75 | 0.56 |
| GDP per capita | Correlation co-efficient | 0.24 | -0.34 |
|  | P-value | 0.29 | 0.13 |
| Maternal education | Correlation co-efficient | 0.36 | 0.37 |
|  | P-value | 0.11 | 0.10 |
| Proportion of births by rural women | Correlation co-efficient | -0.42 | -0.44 |
|  | P-value | 0.06 | 0.04 |
| Rural birth density | Correlation co-efficient | 0.10 | 0.19 |
|  | P-value | 0.68 | 0.40 |
| TFR | Correlation co-efficient | -0.66 | -0.34 |
|  | P-value | <0.01 (0.001) | 0.13 |

| **Table 9) Correlation analysis between contextual and outcome indicators** | | | |
| --- | --- | --- | --- |
| **Contextual indicators** | **Co-efficient & P-value** | **Outcome indicators** | |
|  |  | **Proportion of births by rural women in a health facility** | **Proportion of births by rural women by C-section** |
| Total population | Correlation co-efficient | -0.16 | -0.15 |
|  | P-value | 0.50 | 0.52 |
| GDP per capita | Correlation co-efficient | 0.37 | 0.41 |
|  | P-value | 0.10 | 0.06 |
| Maternal education | Correlation co-efficient | 0.58 | 0.57 |
|  | P-value | 0.01 | 0.01 |
| Proportion of births by rural women | Correlation co-efficient | -0.48 | -0.63 |
|  | P-value | 0.03 | <0.01 (0.0025) |
| Rural birth density | Correlation co-efficient | -0.01 | 0.01 |
|  | P-value | 0.98 | 0.98 |
| TFR | Correlation co-efficient | -0.70 | -0.68 |
|  | P-value | <0.01 (0.0004) | <0.01 (0.0007) |

| **Table 10) Correlation analysis between input and input indicators** | | | | | | | | | | |
| --- | --- | --- | --- | --- | --- | --- | --- | --- | --- | --- |
| **Input indicators** | **Co-efficient & P-value** | **Input indicators** | | | | | | | | |
|  |  | **Per capita recurrent health expenditure** | **OOP expenditure** | **Community health fund coverage** | **ODA per capita to RMNCH** | **Skilled health workforce density by population** | **Skilled health workforce density by births** | **Health facility density by population** | **Health facility density by births** | **% Health facilities with tracer drugs** |
| Per capita recurrent health expenditure | Correlation co-efficient |  | 0.12 | -0.12 | -0.18 | 0.50 | 0.45 | 0.54 | 0.56 | -0.31 |
|  | P-value |  | 0.61 | 0.61 | 0.44 | 0.02 | 0.04 | 0.01 | 0.01 | 0.17 |
| OOP health expenditure | Correlation co-efficient |  |  | -0.40 | -0.43 | 0.42 | 0.51 | 0.37 | 0.50 | -0.10 |
|  | P-value |  |  | 0.08 | 0.05 | 0.06 | 0.02 | 0.10 | 0.02 | 0.67 |
| Community health fund coverage | Correlation co-efficient |  |  |  | 0.18 | 0.03 | 0.04 | -0.19 | -0.06 | -0.04 |
|  | P-value |  |  |  | 0.44 | 0.91 | 0.87 | 0.43 | 0.80 | 0.85 |
| ODA per capita to RMNCH | Correlation co-efficient |  |  |  |  | -0.05 | -0.15 | -0.02 | -0.13 | -0.30 |
|  | P-value |  |  |  |  | 0.85 | 0.51 | 0.93 | 0.56 | 0.19 |
| Skilled health workforce density by population | Correlation co-efficient |  |  |  |  |  | 0.93 | 0.68 | 0.89 | -0.33 |
|  | P-value |  |  |  |  |  | <0.01 (0.000) | <0.01 (0.0006) | <0.01 (0.000) | 0.15 |
| Skilled health workforce density by births | Correlation co-efficient |  |  |  |  |  |  | 0.56 | 0.92 | -0.32 |
|  | P-value |  |  |  |  |  |  | 0.01 | <0.01 (0.000) | 0.16 |
| Health facility density by population | Correlation co-efficient |  |  |  |  |  |  |  | 0.81 | -0.03 |
|  | P-value |  |  |  |  |  |  |  | <0.01 (0.000) | 0.88 |
| Health facility density by births | Correlation co-efficient |  |  |  |  |  |  |  |  | -0.22 |
|  | P-value |  |  |  |  |  |  |  |  | 0.33 |
| % Health facilities with tracer drugs | Correlation co-efficient |  |  |  |  |  |  |  |  |  |
|  | P-value |  |  |  |  |  |  |  |  |  |

| **Table 11) Correlation analysis between contextual and contextual indicators** | | | | | | | |
| --- | --- | --- | --- | --- | --- | --- | --- |
| **Contextual indicators** | **Co-efficient & P-value** | **Contextual indicators** | | | | | |
|  |  | **Total population** | **GDP per capita** | **Maternal education** | **Proportion of births by rural women** | **Rural birth density** | **TFR** |
| Total population | Correlation co-efficient |  | 0.23 | -0.02 | -0.56 | 0.83 | 0.02 |
|  | P-value |  | 0.31 | 0.94 | <0.01 (0.0085) | <0.01 (0.000) | 0.94 |
| GDP per capita | Correlation co-efficient |  |  | 0.66 | -0.64 | 0.43 | -0.64 |
|  | P-value |  |  | <0.01 (0.001) | <0.01 ( 0.0017) | 0.05 | <0.01 (0.001) |
| Maternal education | Correlation co-efficient |  |  |  | -0.50 | 0.23 | -0.67 |
|  | P-value |  |  |  | 0.02 | 0.32 | <0.01 (0.0009) |
| Proportion of births by rural women | Correlation co-efficient |  |  |  |  | -0.79 | 0.50 |
|  | P-value |  |  |  |  | <0.01 (0.0000) | 0.02 |
| Rural birth density | Correlation co-efficient |  |  |  |  |  | -0.21 |
|  | P-value |  |  |  |  |  | 0.36 |
| TFR | Correlation co-efficient |  |  |  |  |  |  |
|  | P-value |  |  |  |  |  |  |

| **Table 12) Correlation analysis between outcome and outcome indicators** | | | |
| --- | --- | --- | --- |
| **Outcome indicators** | **Co-efficient & P-value** | **Outcome indicators** | |
|  |  | **Proportion of births by rural women in a health facility** | **Proportion of births by rural women by C-section** |
| Proportion of births by rural women in a health facility | Correlation co-efficient |  | 0.89 |
|  | P-value |  | <0.01 (0.0000) |
| Proportion of births by rural women by C-Section | Correlation co-efficient |  |  |
|  | P-value |  |  |

| **Table 13) Correlation analysis between output and outcome indicators** | | | |
| --- | --- | --- | --- |
| **Output** | **Co-efficient & P-value** | **Outcome** | |
|  |  | **Proportion of births by rural women in a health facility** | **Proportion of births by rural women by C-section** |
| Women informed of signs of pregnancy complications at ANC | Correlation co-efficient | 0.64 | 0.69 |
|  | P-value | <0.01 (0.0019) | <0.01 (0.0006) |
| Facilities with improved water source | Correlation co-efficient | 0.26 | 0.24 |
|  | P-value | 0.26 | 0.30 |

| **Table 14) Correlation analysis between output and output indicators** | | | |
| --- | --- | --- | --- |
| **Output** | **Co-efficient & P-value** | **Output** | |
|  |  | **Women informed of signs of pregnancy complications at ANC** | **Facilities with improved water source** |
| Women informed of signs of pregnancy complications at ANC | Correlation co-efficient |  | 0.41 |
|  | P-value |  | 0.06 |
| Facilities with improved water source | Correlation co-efficient |  |  |
|  | P-value |  |  |

## References

1. Global Rural-Urban Mapping Project. Version 1 (GRUMPv1): Urban Extents Grid. [Internet]. NASA Socioeconomic Data and Applications Center (SEDAC) <http://dx.doi.org/10.7927/H4GH9FVG>. 2011 [cited 12.08.2015].
2. National Bureau of Statistics of Tanzania, ICF Macro. Tanzania Demographic and Health Survey 2010. Calverton, Maryland: ICF Macro, 2011.
3. Pullum T. Program to produce ASFRs, TFR, GFR for specific windows of time, with covariates. In: Ruktanochai C, editor. 2012
4. Tatem AJ, Campbell J, Guerra-Arias M, de Bernis L, Moran A, Matthews Z. Mapping for maternal and newborn health: the distributions of women of childbearing age, pregnancies and births International Journal of Health Geographics. 2014;13(1)
5. Arregoces L, Daly F, Pitt C, Hsu J, Martinez-Alvarez M, Greco G, et al. Countdown to 2015: changes in official development assistance to reproductive, maternal, newborn, and child health, and assessment of progress between 2003 and 2012. The Lancet Global Health. 2015;3(7):e410-e21
6. The World Bank Food and Agriculture Organization, electronic files and web site. [Internet]. Catalog Sources World Development Indicators – Land Area. <http://data.worldbank.org/indicator/AG.LND.TOTL.K2/countries> . 2016 [cited 13.05.2016]
7. Sikika. Tanzanian Health Sector Budget Analysis 2005/6 - 2011/12. November 2012 Report
8. [Gabrysch S](http://www.ncbi.nlm.nih.gov/pubmed/?term=Gabrysch%20S%5BAuthor%5D&cauthor=true&cauthor_uid=21831117), [Zanger P](http://www.ncbi.nlm.nih.gov/pubmed/?term=Zanger%20P%5BAuthor%5D&cauthor=true&cauthor_uid=21831117), [Campbell OM](http://www.ncbi.nlm.nih.gov/pubmed/?term=Campbell%20OM%5BAuthor%5D&cauthor=true&cauthor_uid=21831117). Emergency obstetric care availability: a critical assessment of the current indicator. [Trop Med Int Health.](http://www.ncbi.nlm.nih.gov/pubmed/21831117) 2012. Jan;17(1):2-8. doi: 10.1111/j.1365-3156.2011.02851. Epub 2011 Aug 11
